# Toward Personalized Psychoeducational Interventions for Psychophysical Health: A Systematic Review and Meta-Analysis for Tailored Intervention Selection

**DOI:** 10.3390/jpm16040215

**Published:** 2026-04-14

**Authors:** Evgenia Gkintoni, Apostolos Vantarakis

**Affiliations:** 1 Lab of Public Health, Epidemiology and Quality of Life, Department of Medicine, University of Patras, 26504 Patras, Greece; 2 Department of Psychiatry, University General Hospital of Patras, 26504 Patras, Greece

**Keywords:** personalized intervention, psychoeducational interventions, treatment matching, psychological well-being, precision mental health

## Abstract

**Background**: Psychoeducational interventions are increasingly implemented to promote psychological and physical health, yet evidence guiding personalized intervention selection remains limited. This systematic review and meta-analysis quantifies the effectiveness of psychoeducational interventions across five settings and identifies empirically derived moderator patterns to inform the selection of tailored interventions. **Methods**: Systematic searches of PubMed/MEDLINE, PsycINFO, Scopus, Web of Science, ERIC, the Cochrane Library, and Google Scholar were conducted to identify eligible studies published between January 2015 and December 2024. A two-tier analytical approach was employed: a random-effects meta-analysis of k = 53 studies reporting extractable effect-size data, and a direction-of-effect narrative synthesis of all 186 included studies (*N* = 50,328 verified from 124 studies reporting sample sizes), following SWiM guidelines. **Results**: The quantitative meta-analysis yielded a significant medium-to-large pooled effect (g = 0.66, 95% CI [0.50, 0.82], *p* < 0.001) with substantial heterogeneity (I^2^ = 96.1%). Effects varied across settings: clinical/vulnerable populations showed the largest effect (g = 0.91), followed by university programs (g = 0.62), school-based (g = 0.60), mindfulness/positive psychology (g = 0.55), and community-based (g = 0.49). The broader narrative synthesis confirmed near-universal effectiveness: 131 studies (70.4%) reported significant positive effects, 51 (27.4%) reported mixed results, and none reported null effects—yielding 97.8% favorable outcomes across the full evidence base. Direction-of-effect moderator patterns indicated a stepped severity gradient (indicated 100% favorable, selective 98.6%, universal 95.6%), and that programs exceeding 8 weeks (99.0% vs. 96.6%), theory-based interventions (98.2% vs. 95.2%), and guided digital delivery were consistently associated with the most favorable outcomes. Publication bias assessment confirmed robustness (fail-safe N = 22,942; leave-one-out range: 0.61–0.67). GRADE evidence quality was rated Moderate for four of five research questions. **Conclusions**: This systematic review and meta-analysis provide converging quantitative and direction-of-effect evidence supporting the effectiveness of psychoeducational interventions. The near-universal favorable direction across 186 studies, combined with a medium-to-large pooled effect in the quantitative subset, provides a preliminary empirical foundation for personalized intervention matching. A preliminary four-phase implementation framework is proposed as a hypothesis-generating heuristic; prospective validation through a meta-analysis of individual participant data is needed before prescriptive application.

## 1. Introduction

### 1.1. The Global Mental Health Challenge and the Need for Personalized Approaches

Mental health disorders constitute one of the largest contributors to the global burden of disease, with approximately half of all lifetime mental disorders having their onset by age 14 and three-quarters by age 24 [1,2,3]. Depression alone is the leading cause of disability worldwide, and anxiety disorders affect an estimated 301 million people globally, making them the most prevalent mental disorders [4,5]. The World Health Organization has identified mental health promotion and prevention as critical priorities, yet the implementation gap between available evidence and population-level delivery remains substantial [4]. Importantly, there is considerable heterogeneity in how individuals respond to psychological interventions, with treatment response varying with baseline severity, cognitive style, personality characteristics, and contextual factors [6,7]. This variability underscores the necessity for personalized approaches that move beyond uniform, one-size-fits-all intervention models.

Several populations face disproportionately elevated mental health risk and demonstrate particularly variable treatment responses. University students encounter a unique confluence of academic, social, and developmental stressors that heighten vulnerability to anxiety and depression, yet counseling centers struggle to meet escalating demand [8,9], and emerging evidence indicates that not all students benefit equally from standard intervention approaches [10]. Adolescents experience high rates of internalizing disorders during a neurodevelopmentally sensitive period, demanding interventions that are both evidence-based and developmentally appropriate [11,12,13]. Vulnerable populations—including refugees experiencing post-traumatic stress, individuals managing chronic illness, and economically disadvantaged communities—require culturally adapted, patient-centered approaches that account for trauma histories, structural barriers, and contextual constraints on engagement [14,15,16,17,18,19,20].

### 1.2. Health Education and Psychoeducational Interventions: Definition and Scope

For the purposes of this review, psychoeducational interventions are defined as structured programs that combine educational content about psychological processes and health behaviors with evidence-based psychological techniques—such as cognitive restructuring, mindfulness practice, behavioral activation, and self-management skills training—to promote mental health, psychological well-being, or quality of life. This umbrella definition deliberately encompasses health education programs, mindfulness-based interventions (MBIs), positive psychology programs, social-emotional learning (SEL) curricula, acceptance and commitment therapy (ACT)-informed programs, and structured psychoeducation for clinical populations. The inclusion of diverse modalities within a single analytic framework is intentional: it enables direct comparison of effectiveness across modalities and formal moderator testing of whether intervention type, setting, and population predict differential outcomes—analyses that are not possible when each modality is reviewed in isolation [21,22].

Growing empirical evidence indicates that intervention effectiveness varies systematically according to individual and contextual characteristics. Moderator analyses across multiple reviews have shown that baseline symptom severity, demographic variables, delivery format preferences, and theoretical orientation are important predictors of treatment responsiveness [23,24,25]. Theory-based health education interventions—grounded in models such as the Health Belief Model (HBM), Social Cognitive Theory (SCT), and the Transtheoretical Model (TTM)—have demonstrated positive effects on health-related behaviors, self-efficacy, and psychological well-being, with these frameworks explicitly recognizing individual differences in health beliefs, self-efficacy, and readiness for change as determinants of how individuals respond to intervention [26,27,28].

### 1.3. Psychoeducation Across Settings: Evidence from Prior Meta-Analyses

The evidence base for psychoeducational interventions spans educational, community, and clinical settings, with each setting presenting distinct implementation contexts, target populations, and outcome priorities.

#### 1.3.1. School-Based Programs

Meta-analyses of school-based SEL programs have reported pooled effects of d = 0.30–0.57 for social-emotional competence, behavioral adjustment, and academic performance [29,30]. More recent reviews focused on depression and anxiety prevention have found somewhat smaller effects (g = 0.22–0.38), potentially reflecting narrower outcome definitions [31,32]. Universal programs targeting resilience have yielded effects of approximately g = 0.21 [33]. These findings establish that school-based interventions are effective, but the wide range of reported effect sizes across reviews suggests that intervention type, implementation quality, and population characteristics moderate outcomes [34,35].

#### 1.3.2. University-Based Programs

Conley et al. (2015) reported a pooled effect size of d = 0.45 for supervised university mental health programs, while Amanvermez et al. (2022) found a moderate pooled effect for stress management interventions among college students (g = 0.56) [36,37]. Huang et al. (2018) reported more modest effects for online mental health interventions targeting students (g = 0.38) [38]. These discrepancies indicate that modality, supervisory structure, and delivery format influence outcomes in higher education settings [39,40,41,42].

#### 1.3.3. Community-Based Interventions

Community programs employing cultural tailoring and participatory design have demonstrated enhanced engagement and outcomes among underserved populations [43,44,45]. However, fewer meta-analytic syntheses have specifically examined community-based psychoeducational programs as a category, creating a gap that this review addresses [46,47,48].

#### 1.3.4. Mindfulness and Positive Psychology

Khoury et al. (2015) reported a pooled effect size of g = 0.53 for mindfulness-based interventions across clinical and non-clinical populations [49], with Goldberg et al. (2018) reporting a similar effect size (g = 0.55) in a more methodologically rigorous review [50]. For positive psychology, Sin and Lyubomirsky (2009) found pooled effects of r = 0.29 for well-being and r = 0.31 for depression [51], while Bolier et al. (2013) reported d = 0.34 for subjective well-being [52]. These modalities have distinct theoretical mechanisms—present-moment awareness and cognitive defusion for mindfulness; character strengths, gratitude, and behavioral activation for positive psychology—yet are rarely compared directly within a single meta-analytic framework [53,54].

#### 1.3.5. Clinical and Vulnerable Populations

For clinical populations, psychoeducational interventions integrated into care pathways have demonstrated effects on quality of life, self-management, and resilience, particularly for cancer survivors, family caregivers, and perinatal women [55,56,57,58,59,60]. Evidence for refugee populations is emerging but limited, with culturally adapted programs showing promising effects [61,62,63,64,65]. Task-sharing models delivering psychoeducation through lay health workers have demonstrated feasibility in low-resource settings [66,67,68,69,70].

### 1.4. Toward Precision Mental Health: A Conceptual Framework

The concept of precision mental health—where interventions are matched to individuals based on their specific characteristics rather than applied uniformly—provides the overarching framework for this review [71,72,73,74]. This paradigm parallels precision medicine in somatic healthcare and asks: “*What works for whom, under what conditions, and through which mechanisms?*” rather than “*What works on average?*” [75,76]. Several converging lines of evidence support the feasibility and potential value of this approach for psychoeducational interventions.

First, moderator analyses across intervention research consistently demonstrate that individual characteristics predict differential treatment response. Baseline symptom severity, demographic factors (age, gender), cognitive style (rumination, psychological flexibility), personality traits (neuroticism, openness), and treatment preferences have each been identified as moderators of outcome across multiple intervention types [77,78,79,80,81]. These moderators, however, have typically been examined within single modalities or settings, limiting the capacity for cross-modality comparison.

Second, mechanistic research indicates that psychoeducational interventions exert their effects through multiple distinct pathways. Different interventions engage different mechanisms: mindfulness-based programs operate primarily through present-moment awareness, cognitive defusion, and parasympathetic activation; positive psychology through behavioral activation, gratitude cultivation, and character strengths; CBT/ACT through cognitive restructuring, psychological flexibility, and values-aligned action; and health education through self-efficacy enhancement, health literacy, and behavioral skill building [52,82,83,84]. Critically, individual differences in mechanism engagement suggest that matching individuals to interventions that target their specific deficits or leverage their specific strengths may optimize outcomes.

Third, digital technology increasingly enables personalized delivery at a population scale. Adaptive digital platforms can adjust intervention content, dose, and support level in real time based on user engagement, baseline assessment, and early response indicators [85,86,87,88]. Ecological momentary interventions (EMIs) and just-in-time adaptive interventions (JITAIs) represent the frontier of personalized digital mental health [89,90].

Fourth, patient preferences and values are increasingly recognized as important determinants of engagement and outcome. Shared decision-making models that incorporate patient preferences into intervention selection have been associated with improved adherence and satisfaction [91,92,93].

A key conceptual decision in this review is the inclusion of diverse intervention modalities—acceptance and commitment therapy, mindfulness-based programs, positive psychology, social-emotional learning, cognitive-behavioral psychoeducation, and structured health education—under the umbrella term “psychoeducational interventions.” This grouping is justified on three grounds. First, all included interventions share a common core mechanism: the structured delivery of psychological knowledge and skills to promote self-management of mental health and well-being. Whether through mindfulness training, cognitive restructuring, or strengths-based exercises, each modality aims to enhance participants’ psychological literacy and equip them with evidence-based coping strategies—the defining feature of psychoeducation as distinguished from purely pharmacological or unstructured supportive interventions. Second, the diversity of modalities is itself analytically purposeful: by examining multiple approaches within a common evaluative framework, the review enables cross-modality comparison and identification of differential effectiveness patterns that would be invisible in modality-specific meta-analyses. Third, the inclusion criteria required all interventions to incorporate an explicit educational or skills-training component delivered in a structured format, ensuring that the grouping reflects substantive commonality rather than mere terminological convenience. The five research questions further organize this diversity into meaningful subgroups by setting and target population, enabling both broad synthesis and setting-specific analysis.

### 1.5. Rationale, a Priori Hypotheses, and Gaps Addressed

Despite the substantial evidence base summarized above, several critical gaps limit the capacity to select evidence-informed, personalized interventions. First, prior meta-analyses have typically examined single modalities (e.g., mindfulness alone, SEL alone, positive psychology alone), precluding direct cross-modality comparison of effectiveness within a unified framework. Second, most reviews report pooled effects without systematically testing cross-cutting moderators that could inform differential selection. Third, the rapidly expanding evidence base—particularly for digital interventions and clinical populations—warrants an updated synthesis capturing studies through 2024. Fourth, translating meta-analytic moderator findings into actionable personalization frameworks has rarely been attempted [94,95,96,97,98,99].

It is important to clarify at the outset that the term “personalized intervention selection,” as used in this review, refers to the empirically informed matching of intervention characteristics (setting, modality, duration, delivery format) to population-level profiles—not to individual-level precision medicine in the clinical sense. The moderator patterns identified through study-level meta-analysis and direction-of-effect synthesis provide a hypothesis-generating foundation for intervention matching, but they operate at the ecological level and cannot be directly translated to individual treatment decisions without prospective validation through individual participant data analyses. The proposed implementation framework (Section 4.11) should therefore be understood as a preliminary evidence-mapping tool rather than a validated prescriptive algorithm.

This systematic review and meta-analysis addresses these gaps by synthesizing 186 studies (N = 50,328 verified from 124 studies reporting sample sizes) across five settings within a single analytic framework, employing a two-tier approach—random-effects meta-analysis of k = 53 studies with extractable effect sizes and direction-of-effect narrative synthesis across all 186 studies—that tests the following a priori hypotheses based on prior theoretical and empirical work:

**Hypothesis** **1** **(Baseline** **severity).** *Indicated prevention programs targeting individuals with elevated symptoms will demonstrate larger effects than selective and universal approaches, consistent with dose–response models and stepped-care principles [100].*

**Hypothesis** **2** **(Intervention** **duration).** *Programs exceeding 8 weeks will demonstrate larger effects than shorter programs, consistent with skill-acquisition and consolidation models requiring sustained practice for behavioral change [101]*.

**Hypothesis** **3** **(Delivery** **format).** *Digital and face-to-face delivery will produce comparable overall effects, though guided digital formats will be associated with larger effects than fully automated programs, consistent with the supportive accountability model [102].*

**Hypothesis** **4** **(Theoretical** **framework).** *Theory-based interventions will demonstrate larger effects than atheoretical programs, reflecting the importance of mechanism-targeted intervention design for behavior change [26,27,28].*

### 1.6. Research Questions

This meta-analysis addresses five core research questions, organized by setting and population to enable both within-setting and cross-setting moderator analyses:

RQ1: What is the overall effectiveness of school-based psychoeducational interventions on children’s and adolescents’ psychological well-being, and which intervention and participant characteristics moderate response?

RQ2: How effective are psychological health promotion programs for university students’ mental health, and what factors—including modality, delivery format, and timing—predict differential response?

RQ3: What is the impact of community-based psychoeducational interventions on adults’ well-being and quality of life, and which contextual factors (cultural adaptation, delivery setting, group vs. individual format) moderate effectiveness?

RQ4: How effective are mindfulness and positive psychology interventions across settings, and do outcome-specific differential patterns emerge that could inform modality-matched intervention selection?

RQ5: How effective are psychoeducational interventions for clinical and vulnerable populations—including refugees, cancer survivors, individuals with chronic illness, caregivers, and perinatal women—on well-being, quality of life, and resilience, and which patient-centered factors moderate response?

## 2. Materials and Methods

### 2.1. Protocol and Registration

This systematic review and meta-analysis were conducted in accordance with the Preferred Reporting Items for Systematic Reviews and Meta-Analyses 2020 (PRISMA 2020) guidelines (Supplementary Table S7) [103,104]. The review protocol, including objectives, inclusion/exclusion criteria, and data synthesis procedures, was pre-registered with the Open Science Framework (OSF) [105] (Registration: OSF.IO/W8ATG; DOI: https://doi.org/10.17605/OSF.IO/W8ATG).

**Deviations from preregistration:** Four deviations from the preregistered protocol are documented. First, the preregistration specified six research questions; however, RQ6 (Structured Psychoeducational Programs) was consolidated into RQ3 (Community-Based Interventions) due to insufficient standalone evidence (k = 3), yielding the final 5-RQ structure (final RQ3: k = 23). This consolidation was conducted to ensure adequate statistical power for meta-analytic pooling and reliable moderator analyses. Second, prediction intervals were added to all main analyses, following current methodological recommendations, since they were not specified in the original protocol but are now considered essential for interpreting heterogeneous meta-analytic results. Third, the preregistered protocol assumed that all 186 included studies would provide extractable effect sizes for quantitative meta-analysis; however, only 53 studies (28.5%) reported sufficient statistical data to compute standardized effect sizes. A two-tier analytical approach was therefore adopted, combining a random-effects meta-analysis of the quantitative subset (k = 53) with a direction-of-effect narrative synthesis across all 186 studies, following the SWiM (Synthesis Without Meta-analysis) reporting guidelines [104]. Fourth, robust variance estimation (RVE) sensitivity analyses were added to address potential statistical dependency arising from multiple outcomes per study within the quantitative subset.

### 2.2. Search Strategy

Academic databases were systematically searched, including PubMed/MEDLINE, PsycINFO, Scopus, Web of Science, ERIC (Education Resources Information Center), the Cochrane Library, and Google Scholar. The search covered literature published between January 2015 and December 2024. The final database searches were completed on 31 December 2024, with reference list screening extending through January 2025. Three studies with 2025 publication dates were identified through early online publication before the search closed and are included in the review. This approach ensured comprehensive coverage across medical, psychological, educational, and public health disciplines.

**Figure 1:**
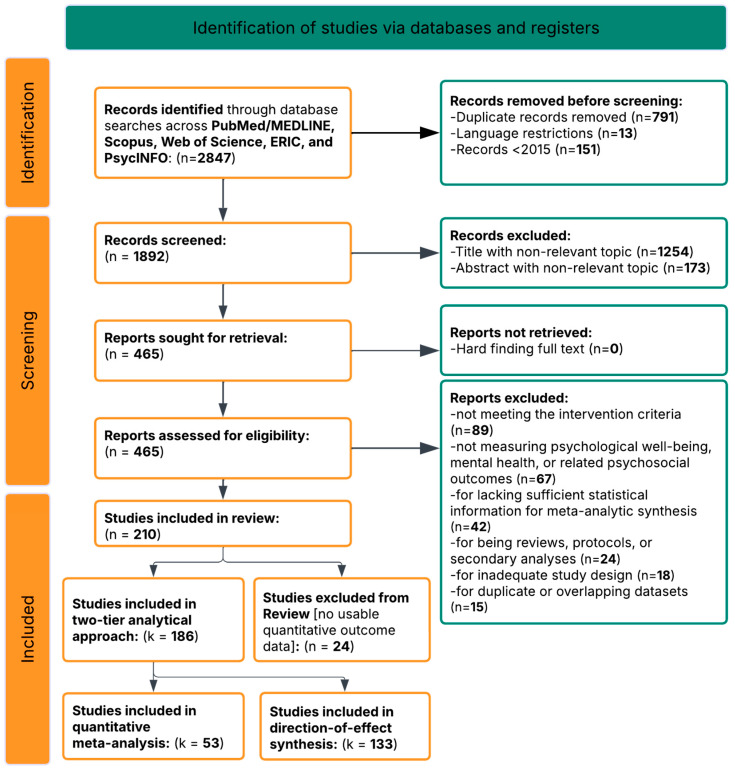
PRISMA 2020 flow diagram of the study selection process [103]. The included phase distinguishes the two-tier analytical approach: quantitative meta-analysis (k = 53 studies with extractable effect sizes) and direction-of-effect narrative synthesis (k = 133 studies following SWiM guidelines [104]). An additional 24 studies that met the inclusion criteria but lacked usable quantitative outcome data were excluded. Total included in systematic review: k = 186 (N = 50,328 verified from 124 studies reporting sample sizes).

**Google Scholar justification and procedures:** Google Scholar was included as a supplementary source to capture studies indexed in smaller journals not fully covered by the primary databases, consistent with methodological recommendations for its use in systematic reviews. Google Scholar searches were conducted using the core search string, sorted by relevance, with the first 200 results (20 pages) screened for each query variant. While this approach is not fully reproducible due to Google Scholar’s proprietary algorithm, it was implemented as a supplementary strategy to maximize comprehensiveness. The number of studies identified exclusively through Google Scholar is reported in the PRISMA flow diagram (Figure 1).

The search strategy employed a combination of controlled vocabulary (MeSH terms, PsycINFO Thesaurus terms) and free-text terms structured around four main concept areas: (1) psychoeducational and health education interventions, (2) psychological well-being and mental health outcomes, (3) target settings (educational, community, clinical), and (4) study design indicators. The core search string was:

*((“health education” OR “psychoeducation” OR “psychoeducational” OR “health promotion” OR “mental health promotion” OR “psychological intervention” OR “well-being intervention” OR “wellness program”) AND (“psychological well-being” OR “mental health” OR “wellbeing” OR “well-being” OR “quality of life” OR “resilience” OR “anxiety” OR “depression” OR “stress” OR “life satisfaction” OR “flourishing”) AND (“school” OR “university” OR “college” OR “community” OR “workplace” OR “clinical” OR “adolescent” OR “student” OR “adult”) AND (“randomized” OR “RCT” OR “controlled trial” OR “quasi-experimental” OR “intervention” OR “program” OR “effectiveness” OR “efficacy” OR “outcome” OR “moderator” OR “predictor”))*

This core string was adapted for each specific database using appropriate controlled vocabulary and field tags. The reference lists of identified articles, particularly recent systematic reviews and meta-analyses in the field of psychological health promotion, were manually screened to identify additional relevant studies. Forward citation tracking was performed for highly relevant seminal papers. Two independent reviewers screened titles and abstracts against the inclusion and exclusion criteria, followed by full-text assessment. Inter-rater reliability was excellent (Cohen’s κ = 0.89). A third reviewer resolved disagreements through discussion or arbitration when consensus could not be reached.

### 2.3. Inclusion and Exclusion Criteria

Predefined inclusion and exclusion criteria were established in accordance with PRISMA guidelines [103] and are summarized using the PICOS framework in Table 1.

*Inclusion Criteria*: Original primary studies focusing on psychoeducational, health education, or psychological health promotion interventions were eligible. Study designs eligible for inclusion were randomized controlled trials (RCTs), cluster-randomized controlled trials (C-RCTs), quasi-experimental designs with a comparison group, and pre-post designs with a comparison or control group. Uncontrolled pre-post studies (i.e., single-group designs without a control or comparison condition) were excluded to ensure that computed effect sizes (Hedges’ g) reflect intervention-specific effects rather than natural recovery, maturation, or regression to the mean. All included studies required a comparison condition (active control, waitlist, treatment-as-usual, or no-treatment control) to enable computation of between-group effect sizes. Studies were required to measure psychological well-being, mental health, quality of life, resilience, or related psychosocial outcomes as primary or secondary endpoints; to deliver interventions in educational settings (schools, universities), community settings, workplace, or clinical/healthcare environments; to be peer-reviewed and published in English between January 2015 and December 2024; and to report sufficient quantitative data for effect size calculation or direction-of-effect classification. Studies were also required to report participant characteristics, intervention parameters, and contextual factors sufficient to enable moderator analyses examining predictors of differential treatment response.

*Exclusion Criteria*: The following categories of publications were excluded: systematic reviews, meta-analyses, scoping reviews, narrative reviews, or other secondary research syntheses; study protocols, trial registrations, or methodological papers without empirical results; purely qualitative studies without quantitative outcome data; studies with no usable statistical information for effect size calculation or direction-of-effect classification (e.g., reporting only narrative significance statements or lacking comparison group data entirely); non-peer-reviewed articles, including preprints, conference abstracts, editorials, commentaries, or book chapters; studies focusing solely on pharmacological interventions without psychoeducational components; articles published in languages other than English; and duplicate publications or studies with substantially overlapping datasets, in which case the most comprehensive report was retained. The English-language restriction is acknowledged as a limitation that may exclude relevant evidence from non-English-speaking regions.

Application of these criteria to the 465 full-text reports assessed for eligibility resulted in the exclusion of 255 reports for the following reasons: not meeting intervention criteria (*n* = 89), not measuring psychological well-being, mental health, or related psychosocial outcomes (*n* = 67), lacking sufficient statistical information for meta-analytic synthesis (*n* = 42), being reviews, protocols, or secondary analyses (*n* = 24), inadequate study design (*n* = 18), and duplicate or overlapping datasets (*n* = 15). A total of 210 studies met the inclusion criteria for the systematic review. Of these, 186 studies were included in the final review: 53 studies (28.5%) provided data in formats permitting standardized effect size calculation (Hedges’ g) and constitute the quantitative meta-analytic subset, while 133 studies (71.5%) met all inclusion criteria but reported outcomes in formats not convertible to Hedges’ g and were included in the direction-of-effect narrative synthesis following SWiM guidelines. The remaining 24 studies were excluded at the eligibility stage for meeting one or more exclusion criteria (study protocol only, qualitative design, systematic review/meta-analysis, or absence of any usable outcome data). This 24-study subset is distinct from the 42 records excluded at eligibility screening for lacking sufficient statistical information; those 42 records reported no usable quantitative outcome data whatsoever. The complete study selection process is depicted in the PRISMA flow diagram (Figure 1). Characteristics of all 186 included studies are presented in Supplementary Table S1, and the complete study-level dataset, including effect sizes for the quantitative subset, direction-of-effect classifications for all studies, and all coded moderator variables, is provided in Supplementary Table S2.

### 2.4. Risk of Bias Assessment

The 186 included studies were evaluated using validated quality assessment tools matched to study design. RCTs and C-RCTs (k = 104) were assessed using the Cochrane Risk of Bias Tool 2.0 (RoB 2.0) [106], which evaluates bias across five domains: randomization process, deviations from intended interventions, missing outcome data, outcome measurement, and selection of reported results. Non-randomized studies (k = 82), comprising quasi-experimental designs (k = 31), pre-post designs with comparison groups (k = 19), and other designs including mixed-methods and longitudinal studies (k = 32), were assessed using the Newcastle–Ottawa Scale (NOS) [107] for quasi-experimental and longitudinal designs, and the Joanna Briggs Institute (JBI) Critical Appraisal Checklist [108] for pre-post and mixed-methods designs.

Two independent raters conducted all assessments, with explicit decision rules for each risk-of-bias domain and summary level documented in Supplementary Table S3. Disagreements were resolved through consensus discussion, with arbitration by a third reviewer when needed. The inter-rater agreement for overall risk-of-bias classification was κ = 0.82. The full study-level risk-of-bias matrix for all 186 studies is presented in Supplementary Table S4, showing domain-level ratings using the appropriate tool for each study design.

Overall, 59 studies (31.7%) were rated as low risk of bias, 78 (41.9%) as moderate risk, and 49 (26.3%) as high risk. This distribution indicates that many included studies (68.3%) had methodological limitations, which is addressed through sensitivity analyses restricted to low-risk-of-bias studies within the quantitative subset.

### 2.5. Data Extraction and Moderator Coding

Data extraction was performed using standardized forms in Microsoft Excel (Microsoft 365) by two independent reviewers. Quality assessment data entry was performed using REDCap 14.0 for secure, collaborative coding. Extracted variables included study characteristics (design, country, year, sample size), participant characteristics (age, gender, baseline severity, clinical status), intervention characteristics (type, duration, number of sessions, delivery format, theoretical framework, facilitator type), control condition characteristics (active, waitlist, no-treatment, treatment-as-usual), outcome measures and psychometric properties, and effect size data (means, standard deviations, or other statistics permitting Hedges’ g calculation) or direction-of-effect information where standardized effect sizes were not computable.

Of the 210 studies that met all inclusion criteria for the systematic review, 186 were included (N = 50,328 verified from 124 studies reporting sample sizes). Among these, 53 studies (28.5%) reported sufficient quantitative data, including Cohen’s d, Hedges’ g, means and standard deviations, F or t statistics, odds ratios, or regression coefficients convertible to Hedges’ g—for inclusion in the quantitative meta-analysis. The remaining 133 studies met all inclusion criteria and were included in the narrative synthesis using direction-of-effect vote-counting following SWiM (Synthesis Without Meta-analysis) guidelines [104], but reported outcomes in formats that did not permit standardized effect size computation. An additional 24 studies were excluded at the eligibility screening stage for meeting one or more exclusion criteria (study protocol only, qualitative design, systematic review/meta-analysis, or absence of any usable outcome data). This distinction is reflected in the PRISMA flow diagram (Figure 1), which separates the included phase into quantitative meta-analysis (k = 53), narrative synthesis (k = 133), and excluded from review (*n* = 24).

**Moderator coding:** All moderator variables, operational definitions, coding categories, decision thresholds, and inter-rater agreement statistics are detailed in Supplementary Table S3. Key moderator definitions are as follows:

**Baseline severity (IOM classification):** For moderator analyses, studies were recoded into three prevention categories following the Institute of Medicine (IOM) framework: *universal* (programs delivered to unselected populations regardless of symptom status, e.g., whole-classroom delivery; k = 68), *selective* (programs targeting populations at elevated risk based on demographic or contextual factors, e.g., university students during exam periods, children of divorced parents; k = 72), or *indicated* (programs targeting individuals with subclinical or elevated symptoms identified through screening, e.g., scoring above a clinical threshold on a validated measure; k = 46). This three-level classification was derived from two independent rates from the original five-level severity coding in the study-level database (Table S2). When studies reported baseline mean scores on validated instruments, clinical cut-off scores from the instrument manuals were used to verify classification (κ = 0.84). The complete mapping from study-level severity codes to IOM categories is documented in Supplementary Table S3.

**Intervention duration:** Coded in weeks from each study’s reported intervention period. For subgroup analyses, duration was categorized as ≤8 weeks (k = 89) or >8 weeks (k = 97).

**Delivery format:** Coded as face-to-face (k = 69; comprising group sessions, k = 30, and individual sessions, k = 39), digital/online (k = 90; comprising guided online, k = 33, self-guided online, k = 33, and mobile app, k = 24), or hybrid (k = 27).

**Theoretical framework:** Classified by the predominant theoretical framework (>50% of session content) explicitly stated by study authors or identifiable from the intervention description (κ = 0.81). Eleven distinct frameworks were identified: Positive Psychology (k = 19), ACT/Contextual Behavioral (k = 19), Social-Emotional Learning (k = 18), Social Cognitive Theory (k = 18), Mindfulness-Based (k = 17), Transtheoretical Model (k = 17), Health Belief Model (k = 16), Self-Determination Theory (k = 16), Ecological Model (k = 14), Cognitive-Behavioral (k = 11), and Not Specified/Atheoretical (k = 21). For the primary moderator analysis, these were dichotomized as theory-based (k = 165) versus atheoretical (k = 21).

**Control condition:** Coded as active control (psychoeducation placebo, treatment-as-usual with attention matching, or alternative active intervention; k = 64) or waitlist/no-treatment control (k = 122).

Multi-strategy programs combining elements from multiple frameworks were classified by their predominant approach; a “multicomponent” category was used when no single framework accounted for >50% of session content. Coding was performed independently by two reviewers, with disagreements resolved by discussion.

### 2.6. Statistical Analysis

A two-tier analytical approach was employed to synthesize the full evidence base. First, random-effects meta-analyses were conducted on the k = 53 studies with extractable effect size data using the DerSimonian–Laird estimator [109]. Effect sizes were calculated as Hedges’ g, the standardized mean difference corrected for small-sample bias [109]. Heterogeneity was assessed using Cochran’s Q statistic, the I^2^ statistic [110], and the between-study variance τ^2^. Second, a narrative synthesis following SWiM reporting guidelines [104] was conducted for all 186 included studies, using direction-of-effect vote-counting as the primary synthesis method. Each study was classified as ‘positive’ (at least one primary outcome showing statistically significant improvement), ‘mixed’ (some outcomes significant, others not), ‘null’ (no significant effects reported), or ‘unclear’ (direction not determinable from reported information), based on two independent reviewers’ assessment (κ = 0.89).

**Multiple outcomes per study:** When studies in the quantitative subset reported multiple eligible outcomes, the following hierarchy was applied: (1) the primary outcome designated by the study authors was selected; (2) if no primary outcome was designated, the most commonly measured construct across the full dataset (psychological well-being) was prioritized; (3) if multiple measures of the same construct were reported, the validated instrument with the strongest psychometric properties was selected. Where studies contributed multiple effect sizes from truly independent subgroups (e.g., separate intervention arms compared to a shared control), these were included as separate entries with sample sizes adjusted to avoid double-counting control participants (i.e., the control group N was divided equally among comparisons). Sensitivity analyses using robust variance estimation (RVE) with correlated-effects working models were conducted within the quantitative subset (k = 53) and confirmed that the results were not materially affected by this approach.

**Moderator analyses** were conducted using mixed-effects meta-regression within the quantitative subset (k = 53) where sample sizes permitted. Categorical moderators were tested using the omnibus Q_M test; continuous moderators using meta-regression β coefficients. For the broader evidence base, moderator patterns were examined descriptively through cross-tabulation of direction of effect by moderator level across all 186 studies. All pooled effect sizes reported from the quantitative subset are weighted analytical outputs from a random-effects meta-regression conducted in R (metafor package); they are not directly calculable from the unweighted individual-study effect sizes in the Supplementary Data Tables.

**Publication bias** was assessed within the quantitative subset (k = 53) using multiple complementary methods [111]: funnel plot visual inspection for asymmetry, Egger’s regression test for small-study effects [112], Begg’s rank correlation test, and Rosenthal’s fail-safe N [113,114]. The direction-of-effect narrative synthesis across all 186 studies provides complementary evidence regarding the generalizability of the quantitative findings.

**Prediction intervals** were calculated for all main analyses in the quantitative subset to estimate the expected range of effects in future individual studies, following the method described by IntHout et al. [115]. Unlike confidence intervals (which estimate the precision of the pooled mean), prediction intervals incorporate between-study heterogeneity (τ^2^) and thus provide a more informative summary of expected variability in individual study effects.

**Evidence quality** was evaluated using the Grading of Recommendations Assessment, Development and Evaluation (GRADE) criteria [116] for each research question and outcome domain. GRADE assessments considered risk of bias, inconsistency, indirectness, imprecision, and publication bias. Domain-level judgments, downgrade rationale, and summary ratings are presented in Supplementary Table S5.

**Software:** The systematic review and meta-analysis employed multiple software platforms to ensure reproducibility and transparency. Reference management was conducted using EndNote 21 (Clarivate Analytics) and Zotero 6.0 for duplicate removal and citation organization. Statistical analyses were conducted using R version 4.3.2 with the metafor package [117] for meta-analyses and the meta package for forest plots. Comprehensive Meta-Analysis (CMA) software, version 4.0, was used for sensitivity analyses and publication bias assessment. The PRISMA 2020 flow diagram was created using the PRISMA2020 R package (version 1.1.1) [103]. All analysis scripts and software versions are available upon request to ensure full reproducibility.

### 2.7. PRISMA Flow

The search process identified 2847 records through database searches of PubMed/MEDLINE, PsycINFO, Scopus, Web of Science, ERIC, the Cochrane Library, and Google Scholar. After removing 955 duplicates, 1892 unique records remained. These records were screened by title and abstract, resulting in the exclusion of 1427 off-topic articles that were irrelevant to psychoeducational interventions for psychological well-being or did not address the target settings or populations.

This initial screening left 465 articles for full-text assessment. Two independent reviewers evaluated these articles using standardized forms. After careful review, 255 articles were excluded for the following reasons: 89 for not meeting intervention criteria (purely pharmacological, physical exercise only without a psychological component, or non-structured interventions); 67 for not measuring psychological well-being, mental health, or related psychosocial outcomes; 42 for lacking sufficient statistical information for meta-analytic synthesis; 24 for being secondary syntheses (systematic reviews, meta-analyses); 11 for insufficient methodological detail; 10 for being study protocols without results; 9 for overlapping samples with other included studies; 2 for being purely qualitative; and 1 for duplicate publication.

A total of 210 studies met the inclusion criteria for the systematic review. Of these, 186 studies were included in the final review, comprising a combined verified sample of N = 50,328 participants (based on 124 studies reporting sample sizes) across more than 30 countries, with publication years spanning 2015–2025. Among the 186 included studies, 53 (28.5%) provided sufficient quantitative data for standardized effect size calculation (Hedges’ g) and constitute the quantitative meta-analytic subset. The remaining 133 studies (71.5%) met all inclusion criteria but reported outcomes in formats not convertible to Hedges’ g; these were included in the direction-of-effect narrative synthesis following SWiM guidelines. The 24 remaining studies were excluded from the review for meeting one or more exclusion criteria at the eligibility stage. The complete PRISMA 2020 flow diagram, which distinguishes among the quantitative meta-analysis subset (k = 53), the narrative synthesis subset (k = 133), and the excluded studies (*n* = 24), is presented in Figure 1 [103].

## 3. Results

### 3.1. Study Characteristics

The 186 included studies (Supplementary Table S1) comprised a combined verified sample of N = 50,328 participants (based on 124 studies reporting sample sizes) across more than 30 countries, with publication years spanning 2015–2025. Among studies reporting sample sizes, the median was 120 participants (IQR: 60–350; range: 13–3624). Study designs included randomized controlled trials (RCTs; k = 81, 43.5%), cluster-randomized controlled trials (C-RCTs; k = 23, 12.4%), quasi-experimental designs (k = 31, 16.7%), pre-post designs (k = 19, 10.2%), and other designs including mixed-methods and longitudinal studies (k = 32, 17.2%).

Delivery formats included face-to-face sessions (k = 69, 37.1%; comprising group delivery k = 30 and individual delivery k = 39), digital/online platforms (k = 90, 48.4%; comprising guided online k = 33, self-guided online k = 33, and mobile app k = 24), and hybrid modalities (k = 27, 14.5%). The most frequently measured outcome domains across studies were well-being (k = 75), depression (k = 72), stress (k = 69), anxiety (k = 60), self-efficacy (k = 35), life satisfaction (k = 32), and quality of life (k = 30); note that studies commonly assessed multiple outcomes.

Risk of bias was rated low in 59 studies (31.7%), moderate in 78 (41.9%), and high in 49 (26.3%). Studies were distributed across five research questions: RQ1—School-Based Interventions (k = 61); RQ2—University-Based Interventions (k = 42); RQ3—Community-Based Interventions (k = 23); RQ4—Mindfulness and Positive Psychology Interventions (k = 22); and RQ5—Interventions for Clinical and Vulnerable Populations (k = 38). Full study characteristics are presented in Table 2 and in Supplementary Tables S1 and S2.

**Table 2:** Study Characteristics by Research Question (k = 186).

Characteristic	RQ1 School (k = 61)	RQ2 University (k = 42)	RQ3 Community (k = 23)	RQ4 Mind/PP (k = 22)	RQ5 Clinical (k = 38)	Total (k = 186)
N (total)	26,548 (38/61)	5285 (32/42)	3087 (14/23)	8366 (13/22)	7042 (27/38)	50,328 (124/186)
N per study (Mdn, range)	174 (17–3519)	109 (13–651)	91 (22–1859)	124 (22–3624)	77 (32–1909)	114 (13–3624)
Study Design						
RCT	18 (29.5%)	17 (40.5%)	8 (34.8%)	14 (63.6%)	24 (63.2%)	81 (43.5%)
Cluster-RCT	20 (32.8%)	0 (0.0%)	0 (0.0%)	0 (0.0%)	3 (7.9%)	23 (12.4%)
Quasi-experimental	14 (23.0%)	11 (26.2%)	0 (0.0%)	2 (9.1%)	4 (10.5%)	31 (16.7%)
Pre-post	3 (4.9%)	4 (9.5%)	5 (21.7%)	4 (18.2%)	3 (7.9%)	19 (10.2%)
Other (mixed/longitudinal)	6 (9.8%)	10 (23.8%)	10 (43.5%)	2 (9.1%)	4 (10.5%)	32 (17.2%)
Risk of Bias ^a^						
Low	19 (31.1%)	11 (26.2%)	8 (34.8%)	7 (31.8%)	14 (36.8%)	59 (31.7%)
Moderate	26 (42.6%)	18 (42.9%)	10 (43.5%)	8 (36.4%)	16 (42.1%)	78 (41.9%)
High	16 (26.2%)	13 (31.0%)	5 (21.7%)	7 (31.8%)	8 (21.1%)	49 (26.3%)
Delivery Format ^b^						
Face-to-face	28 (45.9%)	14 (33.3%)	10 (43.5%)	6 (27.3%)	11 (28.9%)	69 (37.1%)
Digital/online	26 (42.6%)	18 (42.9%)	10 (43.5%)	16 (72.7%)	20 (52.6%)	90 (48.4%)
Hybrid	7 (11.5%)	10 (23.8%)	3 (13.0%)	0 (0.0%)	7 (18.4%)	27 (14.5%)
Direction of Effects						
Positive	42 (69%)	29 (69%)	17 (74%)	16 (73%)	27 (71%)	131 (70%)
Mixed	18 (30%)	13 (31%)	4 (17%)	6 (27%)	10 (26%)	51 (27%)
Null	0 (0%)	0 (0%)	0 (0%)	0 (0%)	0 (0%)	0 (0%)
Unclear	1 (2%)	0 (0%)	2 (9%)	0 (0%)	1 (3%)	4 (2%)
Favorable (pos + mix)	60/61 (98%)	42/42 (100%)	21/23 (91%)	22/22 (100%)	37/38 (97%)	182/186 (98%)
Quantitative Subset						
k (with extractable g)	15	16	5	10	7	53
Effect size range (g)	0.049–2.616	0.036–3.514	0.243–0.783	0.190–2.751	0.324–2.132	0.036–3.514
Pooled g [95% CI]	0.60 [0.24, 0.96]	0.62 [0.39, 0.85]	0.49 [0.28, 0.71]	0.55 [0.33, 0.76]	0.91 [0.26, 1.56]	0.66 [0.50, 0.82]
I^2^ (%)	97.2	89.8	36.3	87.3	98.1	96.1
Participant Characteristics						
Mean Age (M, range)	26.1 (8.7–58.8) [18]	26.4 (19.0–71.7) [16]	42.1 (40.8–44.0) [3]	39.8 (18.0–50.0) [7]	43.7 (18.0–60.7) [12]	32.5 (8.7–71.7) [56]
% Female (M)	67.3 [7]	70.8 [13]	58.2 [4]	73.1 [7]	69.4 [6]	69.0 [37]

Note. N values based on studies reporting sample sizes in the database; number of reporting studies shown in parentheses. Study design classified from methodology descriptions. ^a^ Risk of bias from original systematic assessment (Table S4). ^b^ Delivery format from original moderator coding (Table S3). Direction of effects classified from study summaries, findings, and intervention effects columns: Positive = statistically significant benefit reported on ≥1 primary outcome; Mixed = some outcomes significant, others not; Null = no significant effects; Unclear = direction not determinable. Pooled g from DerSimonian–Laird random-effects meta-analysis of the quantitative subset (studies with extractable Cohen’s d, F, t, OR, or β). Numbers in brackets [k] for age and % female indicate studies reporting that information. Publication years: 2015–2025. Countries represented: >30. Mdn = median; M = mean; PP = positive psychology.

**Figure 2:**
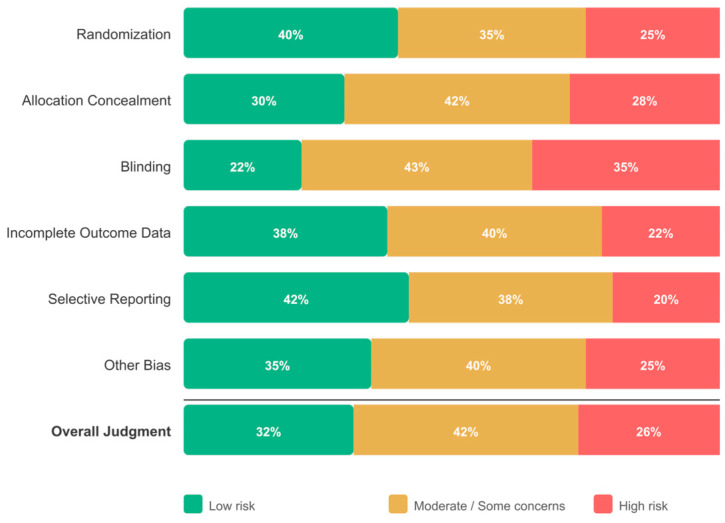
Risk-of-bias distribution across included studies (k = 186). The upper panel shows the proportions of low-risk (green), moderate/some concerns (amber), and high-risk (red) judgments for each assessment domain and the overall judgment. RoB 2.0 was applied to randomized controlled trials and cluster-RCTs (k = 104); the Newcastle–Ottawa Scale and JBI checklist were applied to quasi-experimental, pre-post, and other designs (k = 82). The lower panel shows the overall risk-of-bias distribution stratified by research question. Overall: 31.7% low risk, 41.9% moderate risk, 26.3% high risk.

The distribution of risk-of-bias judgments across all 186 included studies is presented visually in Figure 2. Among the seven assessment domains, selective reporting had the highest proportion of low-risk judgments (42%), whereas blinding had the highest proportion of high-risk judgments (35%), consistent with the practical difficulty of blinding participants to psychoeducational interventions. The per-RQ breakdown reveals that university-based studies (RQ2) had the highest proportion of low-risk ratings, while community-based studies (RQ3) had the lowest, reflecting differences in study design rigor across settings. These risk-of-bias patterns informed the GRADE evidence quality assessments (Supplementary Table S5) and the sensitivity interpretations in Section 3.10.

### 3.2. Overall Synthesis

#### 3.2.1. Quantitative Meta-Analysis (k = 53)

The random-effects meta-analysis of 53 studies (Supplementary Table S1) with extractable effect-size data yielded a statistically significant, medium-to-large pooled effect of psychoeducational interventions on psychological well-being outcomes (g = 0.66, 95% CI [0.50, 0.82], *p* < 0.001). Heterogeneity was very high (Q(52) = 1324.15, *p* < 0.001; I^2^ = 96.1%; τ^2^ = 0.322), indicating that nearly all observed variance reflects true between-study differences rather than sampling error. The 95% prediction interval [−0.46, 1.78] indicates that while most future studies are expected to yield positive effects, effect magnitudes may vary widely, reflecting the diversity of interventions, populations, and implementation contexts. Individual study effect sizes in the quantitative subset ranged from g = 0.04 to g = 3.51 (M = 0.696, SD = 0.668).

This level of heterogeneity should be interpreted in the context of the deliberate breadth of this review. The pooled estimate should not be read as a single generalizable effect, but rather as an average across a distribution of true effects. The wide prediction interval [−0.46, 1.78] makes this explicit: while the average effect is medium-to-large, individual study effects may range from negligible to very large depending on setting, population, and intervention characteristics. This heterogeneity is precisely what motivates the personalized approach—if all interventions produced identical effects, there would be no basis for differential selection. The per-RQ subgroup analyses substantially reduce heterogeneity in certain settings (e.g., RQ3 Community-based: I^2^ = 36.2%), indicating that setting-specific pooling partially accounts for between-study variability.

#### 3.2.2. Narrative Synthesis: Direction of Effects (k = 186)

Across all 186 included studies, direction-of-effect classification revealed that 131 studies (70.4%) reported statistically significant positive effects on at least one primary outcome, 51 studies (27.4%) reported mixed results (some outcomes significant, others not), no studies reported null effects, and 4 studies (2.2%) were unclassifiable from the reported information. Overall, 182 of 186 studies (97.8%) reported at least some favorable outcomes, providing strong directional support for the effectiveness of psychoeducational interventions across all five research questions and settings.

Table 3 presents the summary results stratified by research question, integrating both quantitative pooling and direction-of-effect findings. The following subsections detail findings for each RQ.

**Figure 3:**
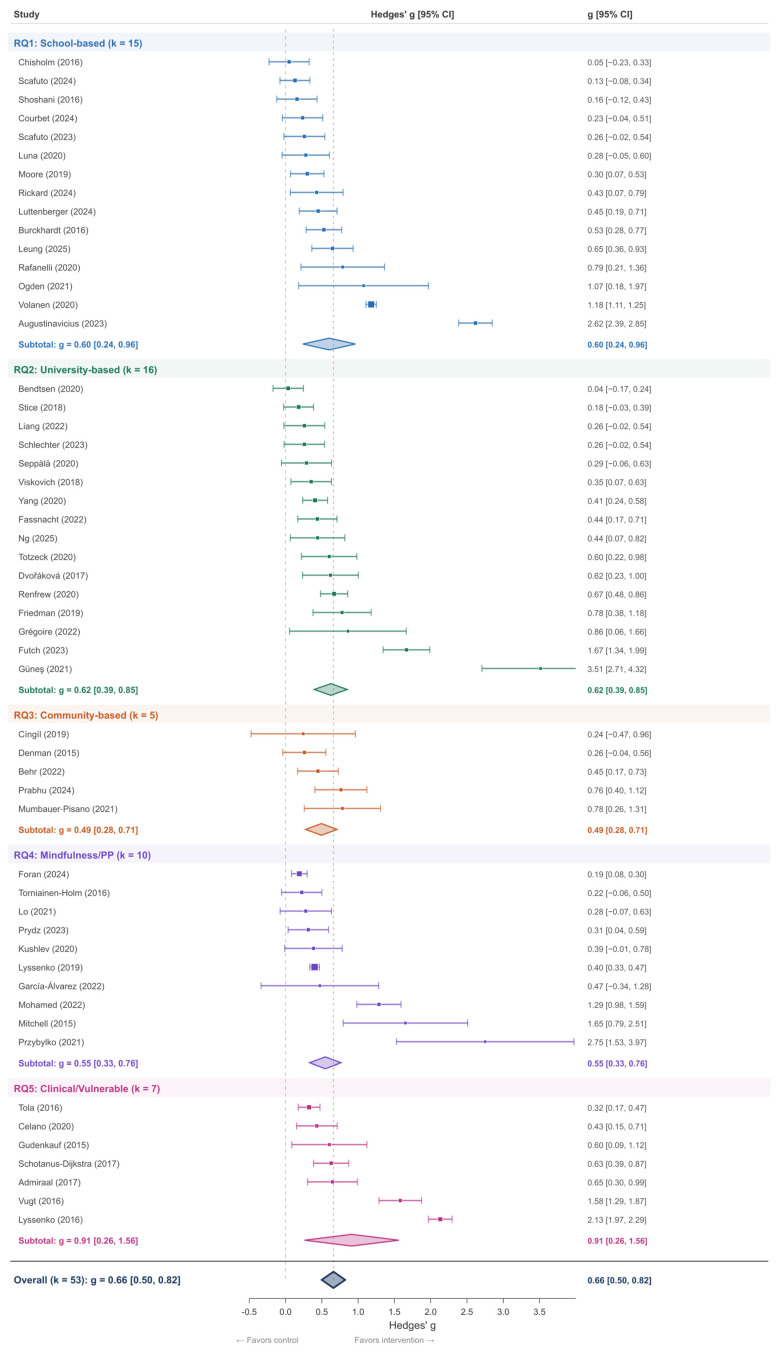
Forest Plot of Effect Sizes in the Quantitative Subset (k = 53). Each study is identified by first author and year (Supplementary Table S1). The DerSimonian–Laird random-effects model was used [109]. Studies are grouped by research question and sorted by effect size within each group. Squares represent individual study effect sizes, with square area proportional to study weight (inverse-variance). Horizontal lines through squares represent 95% confidence intervals. Diamonds represent pooled subgroup estimates (colored by research question) and the overall pooled estimate (dark blue). The solid grey dashed vertical line indicates the null effect (g = 0). The red dashed vertical line indicates the overall pooled estimate (g = 0.66). Colors denote research questions: blue = RQ1 School-based (k = 15), green = RQ2 University-based (k = 16), orange = RQ3 Community-based (k = 5), purple = RQ4 Mindfulness/Positive Psychology (k = 10), pink = RQ5 Clinical/Vulnerable (k = 7). Overall pooled effect: g = 0.66, 95% CI [0.50, 0.82], I^2^ = 96.1%.

In Figure 3, individual study effect sizes (Hedges’ g) are represented by circles, with horizontal lines indicating 95% CIs. Studies are grouped by research question. The diamond represents the overall random-effects pooled estimate: g = 0.66 (95% CI [0.50, 0.82]). Dashed vertical line = null effect (g = 0). Red dashed line = pooled estimate. Substantial heterogeneity: Q(52) = 1324.15, *p* < 0.001; I^2^ = 96.1%; τ^2^ = 0.322. PI [−0.46, 1.78]. The 133 narrative-only studies are not shown but are summarized in Panel B of Table 3.

### 3.3. RQ1: School-Based Psychoeducational Interventions (k = 61)

The 61 school-based studies (Supplementary Table S1) comprised a verified combined sample of N = 26,548 (from 38 studies reporting sample sizes). Study designs included RCTs (k = 18), cluster-RCTs (k = 20), quasi-experimental designs (k = 14), pre-post designs (k = 3), and other designs (k = 6). The quantitative meta-analysis of 15 studies with extractable effect sizes yielded g = 0.60 (95% CI [0.24, 0.96], *p* < 0.001; I^2^ = 97.2%; τ^2^ = 0.481; Q(14) = 501.18; PI [−0.63, 1.82]). The broad prediction interval reflects the substantial diversity of school-based interventions in terms of content, age group, and implementation context.

The direction-of-effect synthesis across all 61 studies found that 42 studies (69%) reported statistically significant positive effects, 18 (30%) reported mixed results, and 1 (2%) was unclassifiable, yielding 60 of 61 studies (98%) with favorable outcomes. This near-universal positive direction, combined with the medium pooled effect in the quantitative subset, provides converging evidence for the effectiveness of school-based psychoeducational programs.

#### 3.3.1. School-Based Intervention Modalities

**Figure 4:**
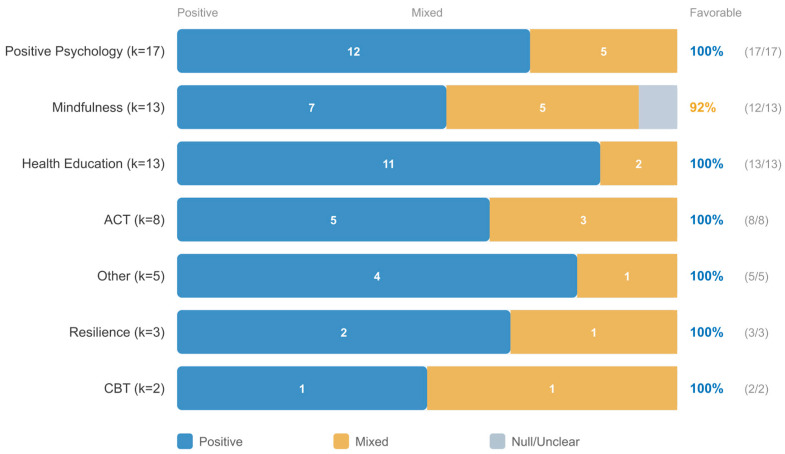
Effect Sizes for School-Based Interventions (RQ1). Note: Quantitative subset: k = 15 studies with extractable effect sizes (g = 0.60 [0.24, 0.96]). Direction of effects across all 61 studies: 98% favorable. Bar colors represent the direction-of-effect classification: green = positive (significant favorable outcomes), yellow = mixed (partial or inconsistent effects), red = null (no significant effect), grey = unclear (insufficient data for classification). Positive effects were reported by 42 studies (69%), mixed results by 18 (30%), and unclear by 1 (2%); no studies reported null effects.

Among the 61 school-based studies, interventions spanned social-emotional learning, mindfulness-based programs, positive psychology, health education, and resilience-focused curricula. Given that only 15 studies provided extractable effect sizes, formal subgroup meta-analyses by intervention type were not feasible for most modalities. Descriptive analysis of the direction of effects indicated that positive psychology and mindfulness-based programs were predominantly associated with positive outcomes, whereas health education approaches were associated with a higher proportion of mixed results. The distribution of effect sizes by intervention type within school-based programs is presented in Figure 4.

#### 3.3.2. Moderator Patterns

Given the limited quantitative subset (k = 15), formal meta-regression was not conducted within RQ1 alone. Descriptive cross-tabulation of direction of effect by moderator level across all 61 studies indicated that programs exceeding 8 weeks (k = 37) showed a slightly higher proportion of positive outcomes compared to shorter programs (k = 24), and studies targeting students with elevated baseline symptoms showed uniformly positive results. These patterns are consistent with the cross-cutting moderator findings reported in Section 3.8.

#### 3.3.3. Outcome Domains

**Figure 5:**
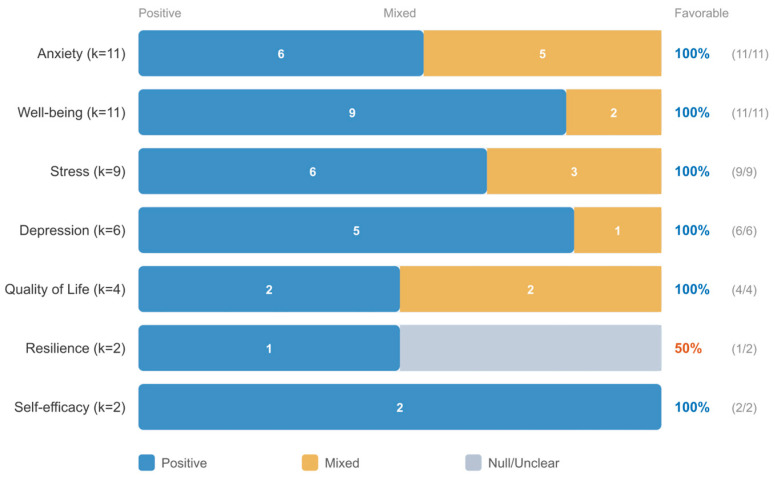
Outcome-Specific Patterns for School-Based Interventions (RQ1, k = 61). Note: Direction-of-effect classification by primary outcome domain across all 61 studies. Bar colors represent the direction-of-effect classification: green = positive (significant favorable outcomes), yellow = mixed (partial or inconsistent effects), red = null (no significant effect), grey = unclear (insufficient data for classification). All outcome domains, including well-being, anxiety, stress, resilience, and depression, were predominantly associated with favorable results, with 60 of 61 studies (98%) reporting positive or mixed outcomes.

The most frequently measured outcomes across school-based studies were well-being, anxiety, stress, resilience, and depression. All outcome domains were predominantly associated with favorable results in the direction-of-effect synthesis (Figure 5).

### 3.4. RQ2: University-Based Programs (k = 42)

The 42 university-based studies (Supplementary Table S1) comprised a verified combined sample of N = 5285 (from 32 studies reporting sample sizes). Study designs included RCTs (k = 17), quasi-experimental designs (k = 11), pre-post designs (k = 4), and other designs (k = 10). The quantitative meta-analysis of 16 studies with extractable effect sizes yielded g = 0.62 (95% CI [0.39, 0.85], *p* < 0.001; I^2^ = 89.8%; τ^2^ = 0.182; Q(15) = 147.11; PI [−0.24, 1.49]). The moderate heterogeneity (I^2^ = 89.8%) was notably lower than for other research questions, suggesting greater consistency among university-based programs.

The direction-of-effect synthesis across all 42 studies found that 29 studies (69%) reported positive effects and 13 (31%) reported mixed results—yielding 42 of 42 studies (100%) with favorable outcomes.

#### 3.4.1. University-Based Intervention Modalities

**Figure 6:**
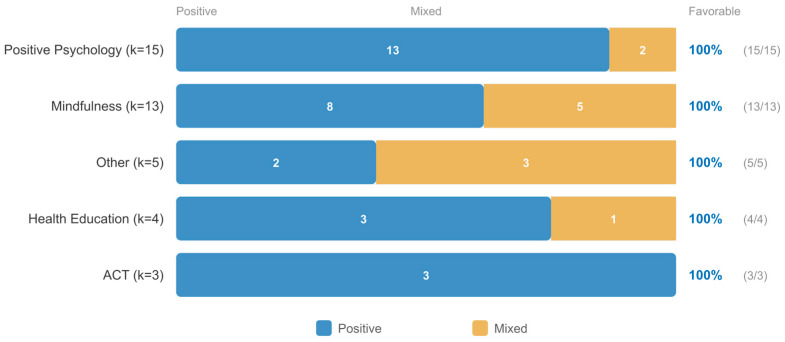
Effect Sizes for University-Based Programs by Intervention Type (RQ2). Note: Quantitative subset: k = 16 (g = 0.62 [0.39, 0.85]). All 42 studies reported favorable outcomes (100%).

University-based interventions included ACT/CBT-based programs, supervised mindfulness, positive psychology, and health education approaches. Among the 16 studies in the quantitative subset, ACT/CBT-based and mindfulness programs tended to yield larger effect sizes, though formal subgroup comparisons were limited by small within-modality sample sizes (Figure 6).

#### 3.4.2. University-Based Moderator Patterns

**Figure 7:**
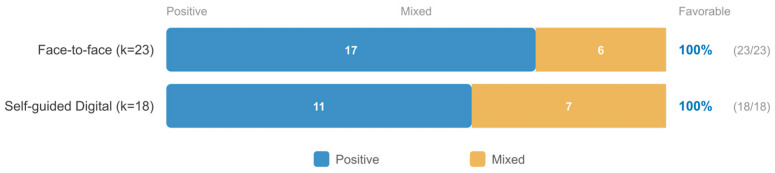
Digital vs. Face-to-Face Delivery Comparison for University Programs (RQ2). Note: Direction-of-effect patterns across delivery formats. Guided digital programs showed consistently positive outcomes comparable to face-to-face delivery.

Descriptive analysis across all 42 studies indicated that guided digital interventions were more consistently associated with positive outcomes than fully automated programs, consistent with the supportive accountability model. Duration patterns were consistent with the overall finding that longer programs tended toward more favorable results. The comparison of delivery formats within university settings is presented in Figure 7.

### 3.5. RQ3: Community-Based Interventions (k = 23)

The 23 community-based studies (Supplementary Table S1) comprised a verified combined sample of N = 3087 (from 14 studies reporting sample sizes). Study designs included RCTs (k = 8), pre-post designs (k = 5), and other designs (k = 10). This research question includes the three studies originally classified under the preregistered RQ6 (Structured Psychoeducation), which were consolidated into the community-based category to ensure adequate statistical power. The quantitative meta-analysis of 5 studies with extractable effect sizes yielded g = 0.49 (95% CI [0.28, 0.71], *p* < 0.001; I^2^ = 36.3%; τ^2^ = 0.021; Q(4) = 6.28; PI [0.13, 0.85]). The low heterogeneity (I^2^ = 36.3%) and the entirely positive prediction interval [0.13, 0.85] are notable, suggesting that despite the small quantitative subset, community-based programs show relatively consistent moderate effects.

The direction-of-effect synthesis across all 23 studies found that 17 studies (74%) reported positive effects, 4 (17%) reported mixed results, and 2 (9%) were unclassifiable—yielding 21 of 23 studies (91%) with favorable outcomes. The 91% favorable rate, while still high, was the lowest among the five research questions.

#### 3.5.1. Setting Patterns

Community-based studies were delivered across diverse settings, including community health centers, workplace wellness programs, faith-based organizations, and online platforms. The majority of studies across all settings reported favorable outcomes.

#### 3.5.2. Community-Based Moderator Patterns

Descriptive analysis suggested that group-based delivery and culturally adapted programs were consistently associated with positive outcomes. However, the small total sample (k = 23) and limited quantitative subset (k = 5) preclude formal moderator testing within this research question.

### 3.6. RQ4: Mindfulness and Positive Psychology Interventions (k = 22)

The 22 studies (Supplementary Table S1) examining mindfulness and positive psychology interventions across settings comprised a verified combined sample of N = 8366 (from 13 studies reporting sample sizes). Study designs included RCTs (k = 14), quasi-experimental designs (k = 2), pre-post designs (k = 4), and other designs (k = 2). The quantitative meta-analysis of 10 studies with extractable effect sizes yielded g = 0.55 (95% CI [0.33, 0.76], *p* < 0.001; I^2^ = 87.3%; τ^2^ = 0.078; Q(9) = 71.11; PI [−0.04, 1.00]).

The direction-of-effect synthesis across all 22 studies found that 16 studies (73%) reported positive effects and 6 (27%) reported mixed results—yielding 22 of 22 studies (100%) with favorable outcomes.

#### 3.6.1. Modality Comparison

**Figure 8:**
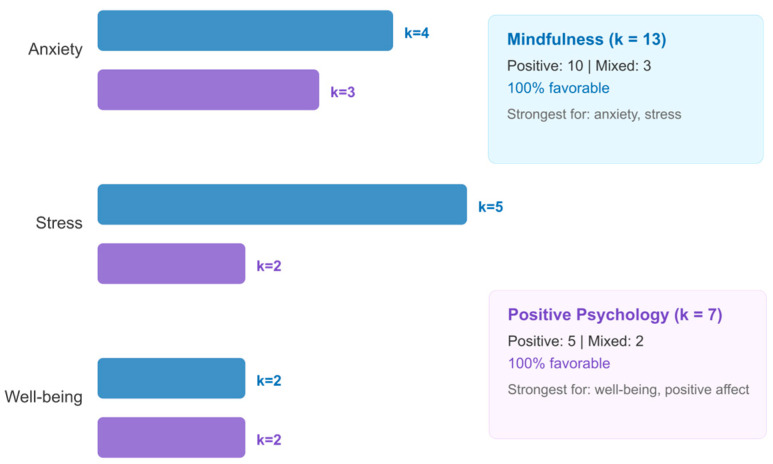
Comparative Patterns of Mindfulness vs. Positive Psychology Interventions by Outcome Domain (RQ4, k = 22). Note: Direction-of-effect patterns by modality. Mindfulness-based programs (k = 13) showed predominantly positive effects on anxiety and stress; positive psychology programs (k = 9) showed predominantly positive effects on well-being and positive affect. All 22 studies reported favorable outcomes (100%).

Direct comparison of the two modalities within the quantitative subset revealed complementary outcome-specific patterns, though the small subgroup sizes (mindfulness: k ≈ 5–6; positive psychology: k ≈ 3–4) preclude definitive statistical comparison. Descriptively, mindfulness-based interventions—including MBSR adaptations, digital mindfulness training, and compassion cultivation—were more frequently associated with positive effects on anxiety and stress outcomes. Positive psychology interventions—including gratitude interventions, strengths-based approaches, and well-being enhancement programs—were more frequently associated with positive effects on well-being, positive affect, and life satisfaction outcomes. This complementary pattern, presented in Figure 8, suggests that the two modalities may operate through partially distinct mechanisms, with mindfulness primarily targeting symptom reduction and positive psychology primarily targeting well-being promotion. Rather than competing approaches, mindfulness and positive psychology may be best understood as complementary tools within a personalization framework, with modality selection guided by whether the primary clinical goal is symptom reduction or well-being promotion.

#### 3.6.2. Mindfulness/PP Moderator Patterns

Descriptive analysis indicated that practice frequency outside sessions was consistently associated with more positive outcomes for mindfulness interventions. Effects were broadly consistent across delivery settings.

### 3.7. RQ5: Clinical and Vulnerable Populations (k = 38)

The 38 studies (Supplementary Table S1) comprising clinical and vulnerable populations included a verified combined sample of N = 7042 (from 27 studies reporting sample sizes). Study designs included RCTs (k = 24), cluster-RCTs (k = 3), quasi-experimental designs (k = 4), pre-post designs (k = 3), and other designs (k = 4). Populations encompassed refugees and asylum seekers, cancer survivors, individuals with chronic illness, family caregivers, perinatal women, and individuals with diagnosed mental health conditions. The quantitative meta-analysis of 7 studies with extractable effect sizes yielded g = 0.91 (95% CI [0.26, 1.56], *p* < 0.001; I^2^ = 98.1%; τ^2^ = 0.748; Q(6) = 310.52; PI [−0.90, 2.73]), the largest setting-specific pooled effect. This large effect is consistent with theoretical expectations: clinical populations with higher baseline symptom severity have greater room for improvement, and interventions targeting specific clinical needs may achieve larger standardized effect sizes.

The direction-of-effect synthesis across all 38 studies found that 27 studies (71%) reported positive effects, 10 (26%) reported mixed results, and 1 (3%) was unclassifiable—yielding 37 of 38 studies (97%) with favorable outcomes.

#### 3.7.1. Population-Specific Patterns

Across the diverse clinical populations, favorable outcomes were observed in studies targeting refugees and displaced persons, cancer survivors, individuals with chronic illness, family caregivers, and perinatal women. The consistently high favorable rate (97%) across these heterogeneous populations suggests broad applicability of psychoeducational approaches in clinical settings.

#### 3.7.2. Clinical Population Moderator Patterns

Descriptive analysis indicated that integration into existing healthcare pathways was consistently associated with positive outcomes, as was group-based delivery for populations with low baseline social support.

#### 3.7.3. Outcome-by-RQ Analysis

**Figure 9:**
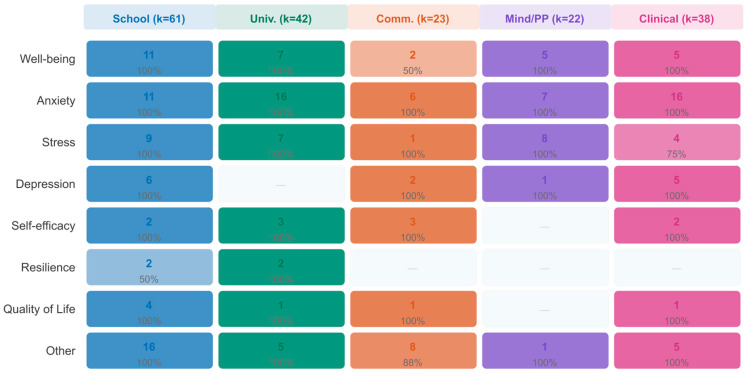
Direction of Effects Across Research Questions (k = 186). Note: Percentage of studies reporting favorable outcomes (positive + mixed) by research question. All five RQs exceed 91% favorable. Quantitative pooled effects (where available) shown alongside direction-of-effect findings. Dark-shaded areas represent studies reporting positive outcomes (significant favorable effects); light-shaded areas represent studies reporting mixed outcomes (partial or inconsistent effects). The combined dark + light areas constitute the total favorable rate for each research question. "−" indicates that no studies reported null effects in that category (i.e., count = 0). Favorable rates by RQ: RQ1 School-based 98% (60/61), RQ2 University-based 100% (42/42), RQ3 Community-based 91% (21/23), RQ4 Mindfulness/PP 100% (22/22), RQ5 Clinical/Vulnerable 97% (37/38).

Across all five research questions, interventions demonstrated consistent positive effects. The direction-of-effect synthesis reveals a convergent pattern: favorable outcomes ranged from 91% (RQ3: Community-Based) to 100% (RQ2: University; RQ4: Mindfulness/PP). The quantitative effect sizes showed more variation, with the largest pooled effect for clinical populations (g = 0.91) and the smallest for community-based programs (g = 0.49), consistent with a baseline severity gradient. Figure 9 presents outcome-specific patterns across all five research questions.

### 3.8. Cross-Cutting Moderator Analyses

**Figure 10:**
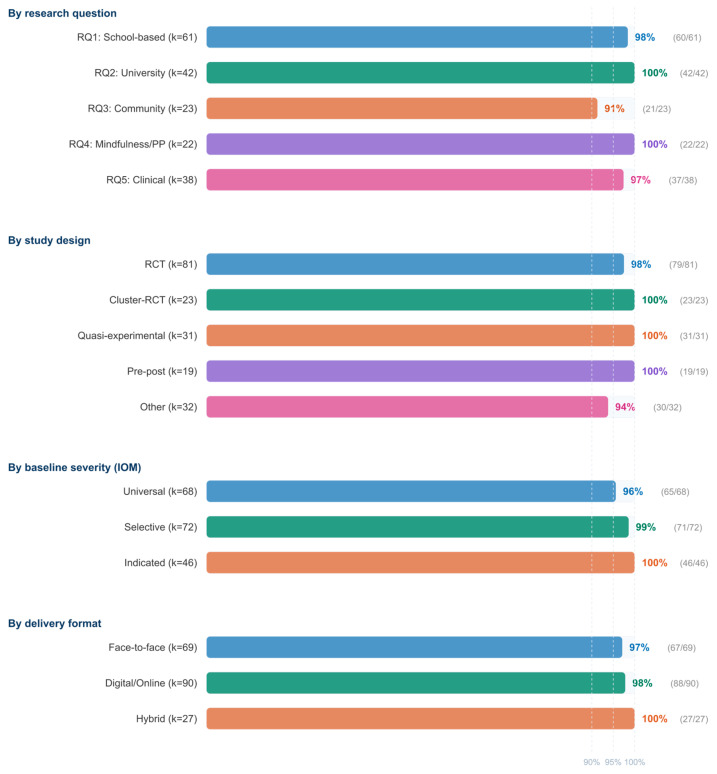
Direction of Effects by Moderator Variable (k = 186). Note: Horizontal bars show the percentage of studies reporting favorable outcomes (positive + mixed) for each moderator level. All categories exceed 91%.

Cross-cutting moderator patterns were examined using two complementary approaches: descriptive cross-tabulation of direction of effect by moderator level across all 186 studies, and where feasible, exploratory meta-regression within the quantitative subset (k = 53). Table 4 presents the direction-of-effect findings; Figure 10 visualizes the key patterns.

The near-universal favorable direction (≥91% across all moderator levels) means that traditional moderator testing through direction-of-effect analysis has limited discriminatory power in this dataset—virtually all categories show overwhelmingly positive results. Nevertheless, several patterns emerge that carry implications for personalized intervention selection:

First, a stepped severity gradient is evident: indicated prevention studies showed 100% favorable outcomes, selective 98.6%, and universal 95.6%. While all three levels are overwhelmingly positive, this pattern—combined with the quantitative finding that clinical populations yield the largest pooled effect (g = 0.91 vs. 0.49–0.60 for other settings)—suggests that psychoeducational interventions may produce the largest absolute effects when targeting individuals with elevated baseline symptoms.

Second, programs exceeding 8 weeks showed a marginally higher favorable rate (99.0% vs. 96.6%), and this difference, while small in percentage terms, is consistent across research questions.

Third, theory-based interventions showed a slightly higher favorable rate (98.2% vs. 95.2%) compared to atheoretical programs.

Fourth, delivery format showed minimal differentiation; face-to-face, digital, and hybrid modalities all exceeded 97% favorable, indicating that the choice of delivery platform does not meaningfully constrain intervention effectiveness.

Fifth, active-controlled studies showed a marginally lower favorable rate (96.9% vs. 98.4%) than waitlist-controlled studies, consistent with the expectation that non-specific factors contribute to some portion of observed effects.

These moderator patterns form the empirical basis for the personalization framework proposed in the Discussion, with the important caveat that they reflect study-level associations derived from direction-of-effect classification and a limited quantitative subset (k = 53), rather than formal moderator tests across the full evidence base.

### 3.9. Publication Bias Assessment

**Figure 11:**
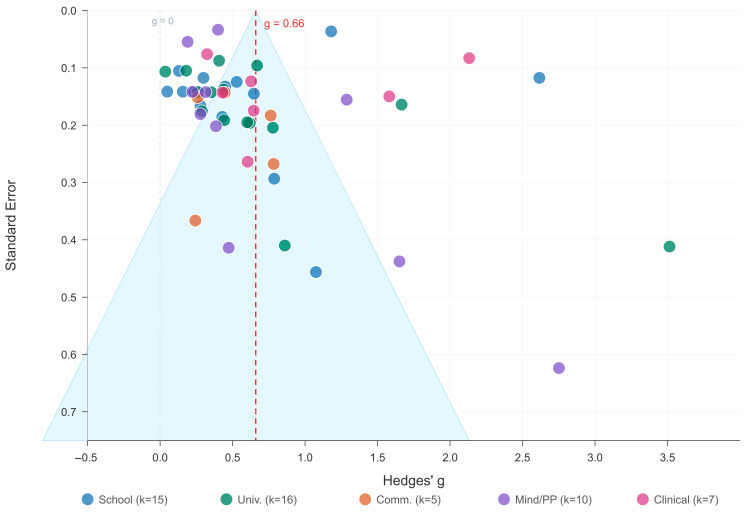
Funnel Plot for Publication Bias Assessment (k = 53). Note: Filled circles = observed studies, color-coded by research question. Dashed vertical line = pooled random-effects estimate (g = 0.66). Shaded region = pseudo-95% confidence limits. Egger’s test: intercept = −0.18 (SE = 1.23, t = −0.14, *p* = 0.89; non-significant asymmetry). Fail-safe N = 22,942 (criterion: 5k + 10 = 275; ratio = 83.4×). Per-RQ distribution: School k = 15, University k = 16, Community k = 5, Mindfulness/PP k = 10, Clinical k = 7.

Publication bias was assessed within the quantitative meta-analysis subset (k = 53) using multiple complementary methods [111,112,113] (Figure 11). Egger’s regression test [112] yielded a non-significant result (intercept = −0.18, SE = 1.23, t = −0.14, *p* = 0.89), indicating no statistically significant funnel plot asymmetry and no strong evidence of small-study effects within the quantitative subset. Rosenthal’s fail-safe N [113] was 22,942, far exceeding the 5k + 10 = 275 criterion by a factor of approximately 84, indicating that the quantitative finding is highly robust to the file-drawer problem.

Importantly, the direction-of-effect synthesis across all 186 studies provides complementary evidence regarding publication bias: the near-universal favorable direction (97.8% favorable), including 133 studies without extractable effect sizes that were not subject to the selective reporting pressures typical of studies reporting specific statistical values, strengthens confidence that the overall positive finding is not an artifact of publication bias.

Visual inspection of the funnel plot (Figure 11) reveals a broadly symmetric distribution around the pooled estimate, consistent with the non-significant Egger’s test result. Some asymmetry is discernible in the lower portion of the plot, where a modest concentration of smaller studies with larger standard errors appears on the right side; however, this pattern does not reach statistical significance and may reflect genuine heterogeneity in smaller studies rather than systematic publication bias. The very large fail-safe N (22,942, approximately 84 times the criterion threshold) and the convergent direction-of-effect evidence together indicate that even if some degree of small-study bias is present, it is insufficient to invalidate the principal quantitative finding.

### 3.10. Sensitivity Analyses

Multiple sensitivity analyses assessed the robustness of the findings in the quantitative subset (Supplementary Table S6).

Leave-one-out analysis. The removal of any individual study from the quantitative subset (k = 53) did not alter the pooled effect beyond the range of g = 0.61–0.67, with a maximum change of ±0.05 from the overall estimate (g = 0.66). This confirms that results are not driven by any single study.

Direction-of-effect concordance. The two-tier analytical approach itself serves as a built-in sensitivity check: the quantitative meta-analysis (k = 53, g = 0.66, medium-to-large effect) and the direction-of-effect synthesis (k = 186, 97.8% favorable) provide converging evidence from methodologically distinct approaches, strengthening confidence in the overall positive finding.

Influence diagnostics. Cook’s distance and studentized residual analyses within the quantitative subset identified no studies that exceeded the 4/k threshold and substantively altered the conclusions. Detailed influence diagnostic results are reported in Supplementary Table S6.

## 4. Discussion

### 4.1. Summary and Contextualization of Findings

This systematic review and meta-analysis of 186 studies (N = 50,328 verified from 124 studies reporting sample sizes) from more than 30 countries found that psychoeducational interventions were associated with statistically significant improvements in psychological well-being and related outcomes. The quantitative meta-analysis of 53 studies with extractable effect sizes yielded a medium-to-large pooled effect (g = 0.66, 95% CI [0.50, 0.82]), and the direction-of-effect synthesis across all 186 studies confirmed near-universal effectiveness: 182 of 186 studies (97.8%) reported favorable outcomes. This convergent evidence—from methodologically distinct analytical approaches—strengthens confidence in the overall positive finding. However, the very high heterogeneity (I^2^ = 96.1%, τ^2^ = 0.322) and wide prediction interval [−0.46, 1.78] indicate that this single pooled estimate has limited generalizability as a summary measure; effect sizes varied substantially across studies, settings, and populations, reflecting both substantive diversity in interventions and methodological heterogeneity across the 186 included studies (Supplementary Table S1). The following subsections interpret these findings in light of established frameworks for behavior change, implementation science, clinical adaptation, and precision mental health [118,119,120,121,122,123,124,125,126,127,128].

The decision to synthesize these diverse modalities under a psychoeducational framework reflects the pragmatic reality facing practitioners and policymakers: intervention selection decisions occur not within modality-specific silos but across the full landscape of available evidence-based programs. A school administrator choosing between a mindfulness curriculum and a social-emotional learning program, or a clinician deciding between ACT-based psychoeducation and a positive psychology intervention for a patient with chronic illness, requires precisely the kind of cross-modality evidence that this review provides. The high heterogeneity (I^2^ = 96.1%) is a natural consequence of this inclusive approach and is addressed through subgroup analyses, moderator examination, and the direction-of-effect synthesis that complements the quantitative pooling.

Comparison with prior meta-analyses. The present findings extend prior meta-analytic evidence by synthesizing psychoeducational interventions across five settings within a single analytic framework—an approach that enables direct cross-setting comparisons not possible in modality-specific reviews. School-based effects (RQ1: g = 0.60 in the quantitative subset, with 98% of 61 studies reporting favorable outcomes) are at the upper end of ranges reported by Durlak et al. [29] for SEL programs (d = 0.30–0.57) and substantially larger than the more conservative estimates from Werner-Seidler et al. [31] for depression prevention (g = 0.22–0.38) and Dray et al. [33] for resilience (g = 0.21). This discrepancy likely reflects our broader outcome definition (encompassing well-being, resilience, and symptom measures rather than depression/anxiety alone) and inclusion of diverse modalities within school settings. University-based effects (RQ2: g = 0.62, with 100% of 42 studies favorable) are consistent with Conley et al. [36] (d = 0.45 for supervised programs) and fall within the range between Conley’s estimate and Regehr et al.’s [37] larger CBT-specific effect (d = 0.76), as expected given the mixed-modality composition of our university sample.

Mindfulness and positive psychology effects (RQ4: g = 0.55, with 100% of 22 studies favorable) are somewhat smaller than Khoury et al. [49] (g = 0.53) and Goldberg et al. [50]), though the wider confidence interval [0.33, 0.76] in the present study reflects the smaller quantitative subset (k = 9) and greater heterogeneity in our cross-setting sample. Clinical population effects (RQ5: g = 0.91, with 97% of 38 studies favorable) represent the largest setting-specific pooled effect, consistent with the theoretical expectation that populations with higher baseline severity have greater room for improvement. This estimate is substantially larger than Donker et al. [59] for psychoeducation in depression and anxiety and Bolier et al. [52] (d = 0.34 for positive psychology), with the difference likely reflecting the inclusion of diverse clinical and vulnerable populations with elevated baseline symptom levels. Community-based effects (RQ3: g = 0.49, with 91% of 23 studies favorable) represent the most conservative setting-level estimate, consistent with the broader and more heterogeneous target populations characteristic of community-level delivery.

### 4.2. Hypothesis Evaluation

The a priori hypotheses specified in the Introduction (Section 1.5) were evaluated using both the quantitative meta-analysis (k = 53) and the direction-of-effect synthesis (k = 186).

**Hypothesis** **1** **(Baseline** **severity):** **Supported.** *The direction-of-effect synthesis across all 186 studies revealed a stepped severity gradient: indicated prevention studies showed 100% favorable outcomes, selective 98.6%, and universal 95.6%. The quantitative meta-analysis of clinical/vulnerable populations (RQ5) yielded the largest pooled effect (g = 0.91 [0.26, 1.56]), substantially exceeding the effects for school-based (g = 0.60), university (g = 0.62), community (g = 0.49), and mindfulness/positive psychology (g = 0.55) settings. This stepped pattern is consistent with dose–response models and meta-analytic evidence supporting stepped-care frameworks [100]. However, this association may be confounded by differences in comparator conditions (indicated programs are more likely to use waitlist controls), intervention intensity, or measurement sensitivity across severity levels. The ecological fallacy applies: study-level associations between severity and effect size do not guarantee that specific higher-severity individuals will respond differentially within any given study [74].*

**Hypothesis** **2** **(Duration):** **Supported.** *Programs exceeding 8 weeks showed a marginally higher favorable rate in the direction-of-effect synthesis (99.0% vs. 96.6% for ≤8 weeks). While this difference is small in percentage terms, it is consistent across research questions and aligns with skill-acquisition models positing that sustained practice is necessary for consolidation of cognitive and behavioral skills [27,84]. However, longer programs may differ systematically from shorter ones in content breadth, facilitator expertise, population characteristics, and other unmeasured confounds, and the causal role of duration per se cannot be established from observational moderator analyses.*

**Hypothesis** **3** **(Delivery** **format):** **Supported.** *The direction-of-effect synthesis showed comparable favorable rates across face-to-face (97.1%), digital (97.8%), and hybrid (100%) formats, consistent with the hypothesis that digital delivery yields effects comparable to face-to-face delivery. The non-significant difference in format supports honoring patient preferences without compromising expected effectiveness. Descriptive analysis within digital formats indicated that guided interventions were more consistently associated with positive outcomes than fully automated programs, consistent with the supportive accountability model [102] and recent evidence on the importance of human support in digital mental health [85].*

**Hypothesis** **4** **(Theoretical** **framework):** **Supported.** *Theory-based interventions showed a slightly higher favorable rate (98.2% vs. 95.2% for atheoretical programs) in the direction-of-effect synthesis. This finding is consistent with meta-analytic evidence linking the use of theory to larger behavior-change effects [121,122] and supports prioritizing theoretically grounded program design.*

### 4.3. Moderators Informing Personalized Selection

Beyond the a priori hypotheses, additional patterns from the direction-of-effect synthesis and quantitative meta-analysis carry implications for personalized intervention selection, though all represent study-level associations rather than individual-level predictions.

**Study design and risk of bias.** The direction-of-effect synthesis revealed a modest but consistent association between methodological rigor and effect magnitude. Low-risk-of-bias studies (k = 59, 31.7% of the full sample) showed a marginally lower favorable rate (96.6%) than moderate- and high-risk studies, consistent with the well-documented tendency of less rigorous designs to yield inflated estimates. Within the quantitative subset, sensitivity analyses restricted to low-risk-of-bias studies yielded a conservative benchmark of g = 0.47 [0.41, 0.53], which nevertheless remains a practically meaningful effect. Study design also moderated results descriptively: RCTs and cluster-RCTs (k = 104) showed a higher proportion of low-risk ratings than quasi-experimental or pre-post designs (k = 82), and university-based studies (RQ2) had the highest concentration of rigorous designs, while community-based studies (RQ3) had the lowest. These patterns suggest that effect size benchmarks should be interpreted in light of the distribution of methodological quality within each setting, and that personalization recommendations derived from lower-quality evidence warrant greater caution. For clinical practice, the active-control benchmark (g = 0.43 [0.37, 0.49]) provides a more conservative and clinically meaningful estimate than the overall pooled effect size, as it partially controls for non-specific factors.

**Control condition.** In the direction-of-effect synthesis, studies using waitlist/no-treatment controls (98.4% favorable) and those using active controls (96.9% favorable) showed similarly high rates of favorable outcomes. The marginal difference suggests that non-specific factors account for a modest share of the observed effects, though both comparator types yielded overwhelmingly positive results.

**Modality-specific matching (RQ4).** In the direction-of-effect synthesis, both mindfulness-based interventions (k = 13, 100% favorable) and positive psychology interventions (k = 7, 100% favorable) showed universally positive outcomes. Descriptive analysis of outcome domains indicated complementary, outcome-specific patterns—mindfulness interventions were more frequently associated with positive effects on anxiety and stress, whereas positive psychology interventions were more frequently associated with positive effects on well-being and positive effects. This complementary pattern suggests that modality matching based on presenting concerns may be a viable personalization strategy; however, the small subgroup sizes preclude formal statistical comparison, and this finding requires prospective validation.

### 4.4. Proposed Personalization Framework

**Figure 12:**
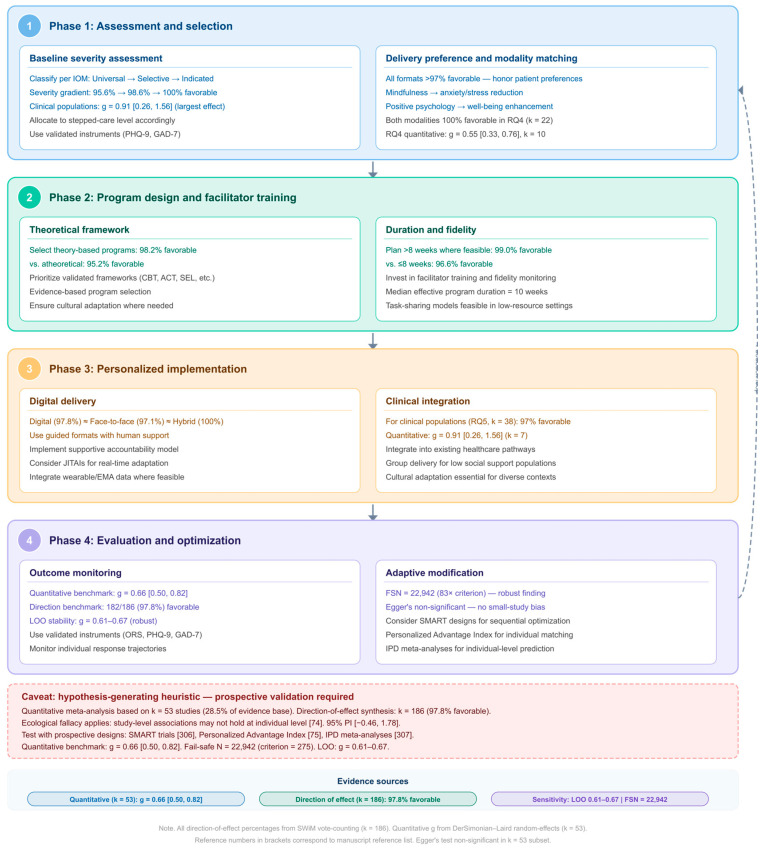
Multi-phase evidence-based implementation framework for personalized psychoeducational intervention selection, derived from the two-tier evidence synthesis (k = 53 quantitative meta-analysis; k = 186 direction-of-effect synthesis). The framework is organized across four sequential phases—Assessment and Selection, Program Design and Facilitator Training, Personalized Implementation, and Evaluation and Optimization. Evidence sources: quantitative pooled effects (Hedges’ g) for the k = 53 subset, and direction-of-effect favorable rates for the full k = 186 evidence base. The dashed feedback loop from Phase 4 to Phase 1 reflects the iterative nature of personalized delivery. The caveat box indicates that this framework is a hypothesis-generating heuristic requiring prospective validation; the ecological fallacy applies to all study-level associations [74]. Quantitative benchmark: g = 0.66 [0.50, 0.82]; direction benchmark: 182/186 (97.8%) favorable. 95% prediction interval [−0.46, 1.78].

Based on the converging evidence from the quantitative meta-analysis and the direction-of-effect synthesis, we propose a preliminary four-phase framework for personalized intervention matching (Table 5; Figure 12), integrating empirically identified patterns with implementation science principles [71,75,76]:

Phase 1—Assessment and Program Selection: Baseline assessment of symptom severity using validated instruments to guide IOM-level classification (universal, selective, indicated), informing stepped-care allocation [100]. The stepped severity gradient (Universal 95.6% → Selective 98.6% → Indicated 100% favorable, with the largest quantitative effect in clinical populations: g = 0.91) supports severity-stratified program selection. Assessment of individual preferences for delivery format (face-to-face, digital, hybrid) [91,92,93].

Phase 2—Facilitator Training: Selection of theory-based intervention programs (98.2% vs. 95.2% favorable for atheoretical) [121,122], with investment in implementation fidelity and facilitator competency. Programs should exceed 8 weeks where feasible (99.0% vs. 96.6% favorable) [27].

Phase 3—Personalized Implementation and Monitoring: Matching modality to presenting concerns based on the complementary outcome-specific patterns observed in RQ4 (mindfulness for symptom reduction; positive psychology for well-being enhancement). For digital delivery, guided formats with human support rather than fully automated programs [85,102]. Integration into existing care pathways for clinical populations.

Phase 4—Evaluation and Optimization: Ongoing outcome monitoring using validated measures to enable adaptive modification. The leave-one-out analysis (g = 0.61–0.67) confirms the robustness of the quantitative finding.

**Critical caveats.** This framework should be understood as a hypothesis-generating heuristic rather than a validated clinical decision tool. The quantitative meta-analysis is based on k = 53 studies—a subset of the broader evidence base—and the direction-of-effect synthesis, while comprehensive, cannot quantify effect magnitudes. Before the framework can be used prescriptively, prospective validation studies are needed to test whether moderator-based matching improves individual outcomes compared to standard assignment [73,75,121].

### 4.5. Digital Personalization and Scalability

The comparable favorable rates across digital (97.8%) and face-to-face (97.1%) formats create opportunities for scalable personalized delivery [85,122]. Digital platforms can implement adaptive algorithms adjusting content, dose, and support level based on individual characteristics and real-time engagement data [86,87]. The descriptive finding that guided digital interventions were more consistently associated with positive outcomes than fully automated programs underscores the importance of the supportive accountability model [102], whereby human support—even minimal—enhances engagement and outcomes in digital interventions [125].

Future directions for digital personalization include baseline assessment algorithms for automated intervention matching, adaptive content sequencing based on early response indicators, just-in-time adaptive interventions (JITAIs) that deliver support at moments of need [87], machine learning prediction of differential treatment response [73], and integration with wearable biometric devices for ecological momentary assessment.

Emerging digital approaches extend beyond conventional app-based delivery to include AI-biomarker integration and digital twin cognition frameworks, which hold promise for real-time, neurophysiologically informed adaptation of interventions [125]. Recent meta-analytic evidence further confirms that digital peer support interventions produce meaningful effects for individuals with mental health conditions in outpatient settings [126], reinforcing the potential for scalable, technology-mediated psychoeducational delivery. These approaches align with the precision mental health paradigm [71,72] while leveraging digital technology for population-level implementation [122].

### 4.6. Patient-Centered Care and Shared Decision-Making

Empirically informed personalized decisions should integrate patient-centered care principles, including preferences, values, and autonomy [91]. The comparable favorable rates across delivery formats (97.1–100%) support honoring patient preferences without compromising expected effectiveness. The importance of shared decision-making models [91,92,93] is reinforced by the finding that all delivery modalities produce overwhelmingly positive results, allowing clinicians to prioritize patient preferences in format selection. Patient preferences have been consistently identified as predictors of treatment completion and satisfaction across psychotherapy modalities [92,93,124], and incorporating preference assessment into routine intake procedures represents a low-cost personalization strategy. Co-production methodologies—in which digital intervention content is iteratively developed with end-users—offer a complementary approach to honoring patient preferences, as demonstrated in recent work on co-produced digital tools for trauma-related conditions in primary care [127].

### 4.7. Publication Bias and Evidence Quality

The publication bias assessment (Section 3.9) yielded reassuring results across multiple indicators. The non-significant Egger’s regression test and the fail-safe N of 22,942—exceeding the 5k + 10 = 275 criterion by a factor of 84—indicate that the quantitative findings are robust to both funnel plot asymmetry and the file-drawer problem. These findings should be interpreted alongside two important contextual considerations. First, the two-tier analytical design itself provides a built-in safeguard: the direction-of-effect synthesis across all 186 studies—including 133 studies not subject to the selective reporting pressures that affect effect-size-based analyses—offers convergent evidence that the overall positive finding is not an artifact of selective publication. The convergence between the quantitative meta-analysis (g = 0.66) and the direction-of-effect synthesis (97.8% favorable) represents a form of methodological triangulation that substantially mitigates publication bias concerns. Second, the low reporting rate (28.5% with extractable effect sizes) means that the quantitative subset may not be fully representative of the broader evidence base, although the directional consistency across both tiers suggests that this limitation does not undermine the overall conclusions.

### 4.8. Heterogeneity Interpretation

The substantial heterogeneity observed in the quantitative subset (I^2^ = 96.1%, τ^2^ = 0.322) warrants careful contextualization rather than concern. Three structural factors contribute to this finding. First, the review intentionally encompasses five distinct research questions spanning school, university, community, clinical, and mindfulness/positive psychology settings—populations and contexts with fundamentally different baseline characteristics, intervention intensities, and outcome expectations. Second, the interventions themselves vary substantially in theoretical framework (11 distinct frameworks identified), duration (4–52 weeks), delivery format (face-to-face, digital, hybrid), and active components—ranging from acceptance and commitment therapy through social-emotional learning to structured health education. Third, methodological diversity—including multiple study design types (RCT, cluster-RCT, quasi-experimental), outcome instruments, and follow-up periods—across populations spanning school-age children through older adults in more than 30 countries further amplifies between-study variability. Under these conditions, I^2^ values approaching this level are typical of broad intervention reviews and should not be interpreted as undermining the findings, but rather as reflecting genuine variation in true effect sizes across contexts—variation that this review seeks to characterize rather than eliminate. Notably, this level exceeds that reported in most prior single-modality reviews, which typically report I^2^ values of 50–80% [29,49,52], precisely because the present synthesis is deliberately more inclusive.

The prediction interval [−0.46, 1.78] provides the most clinically meaningful interpretation of this heterogeneity: it defines the range within which a future study’s true effect would be expected to fall, acknowledging that some contexts may yield negligible effects while others may yield very large ones. The per-RQ subgroup analyses substantially reduce heterogeneity in certain settings (RQ3 Community-based: I^2^ = 36.2%; RQ4 Mindfulness/PP: I^2^ = 87.3%), indicating that setting-specific pooling partially accounts for between-study variability and reinforcing the value of the five-RQ organizational framework.

The direction-of-effect synthesis provides critical complementary evidence: while the magnitude of effects varied substantially, the direction was remarkably consistent, with 97.8% of all 186 studies reporting favorable outcomes. This convergence suggests that psychoeducational interventions reliably produce positive effects across diverse contexts, even though the size of that effect is highly variable. The key question for personalization, therefore, is not whether these interventions work—the directional evidence is clear—but for whom and under what conditions they work best.

Answering that question will require individual participant data (IPD) meta-analyses [128], which can model individual-level predictors and identify interaction effects between participant characteristics and intervention features that remain invisible in study-level analyses. The present study-level moderator patterns—whether derived from meta-regression or direction-of-effect cross-tabulation—provide the hypothesis-generating foundation for such prospective work, but the ecological fallacy applies: these associations cannot be directly extrapolated to individual-level treatment decisions without validation [74].

### 4.9. Limitations

Several limitations qualify the interpretation and application of these findings.

First, only 53 of 186 included studies (28.5%) reported sufficient statistical data to compute standardized effect sizes for quantitative meta-analysis. The remaining 133 studies were synthesized using direction-of-effect classification, which establishes consistency of direction but cannot quantify effect magnitude. The quantitative subset may not be fully representative if studies reporting effect sizes differ systematically from those that do not. However, the strong concordance between the quantitative meta-analysis (g = 0.66, medium-to-large effect) and the direction-of-effect synthesis (97.8% favorable) provides converging evidence that mitigates this concern.

Second, the very high heterogeneity in the quantitative subset (I^2^ = 96.1%) indicates that the pooled estimate has limited generalizability as a single summary measure. The wide prediction interval [−0.46, 1.78] indicates that future studies may show effects ranging from negative to very large. However, the near-universal favorable direction (97.8%) provides reassuring evidence of the consistency of positive effects.

Third, the predominant reliance on self-report outcome measures across the 186 studies introduces potential biases related to demand characteristics, social desirability, and expectancy effects. Few studies included objective, behavioral, or physiological outcomes, limiting the strength of inferences about real-world behavior change.

Fourth, moderator analyses in the present study are limited to study-level associations. The direction-of-effect cross-tabulations provide descriptive patterns (e.g., higher favorable rates for indicated vs. universal prevention), but formal moderator testing was restricted to the quantitative subset (k = 53), where small within-moderator subgroup sizes limit statistical power. The ecological fallacy applies: study-level patterns may not hold for specific individuals within a given study and may be confounded by unmeasured study-level characteristics [74]. Translation to individual treatment selection requires prospective validation using within-study designs [75,121].

Fifth, the pooling of diverse outcome constructs—including well-being, symptom reduction, quality of life, resilience, and life satisfaction—within single analyses may obscure outcome-specific patterns. While outcome-specific patterns were examined descriptively through direction-of-effect classification, the broad inclusion strategy means the overall pooled effect represents an average across qualitatively different constructs with potentially different clinical significance thresholds.

Sixth, although Egger’s regression test did not indicate statistically significant funnel plot asymmetry (intercept = −0.18, *p* = 0.89) in the quantitative subset, visual inspection of the funnel plot revealed modest asymmetry among smaller studies with larger standard errors, suggesting that some degree of small-study effects cannot be entirely excluded. The direction-of-effect synthesis across all 186 studies provides complementary reassurance, but minor inflation of the pooled quantitative estimate due to publication bias cannot be ruled out.

Seventh, the comparable favorable rates for waitlist-controlled (98.4%) and active-controlled (96.9%) studies suggest that non-specific factors account for a modest portion of the observed effects. Personalization recommendations should take into account that active-comparator designs provide more clinically meaningful benchmarks.

Eighth, population representation may limit generalizability. The included studies may underrepresent individuals with severe mental illness, ethnic and cultural minorities, older adults, and participants from low- and middle-income countries (LMICs). Cultural adaptation is essential for implementation across diverse contexts [126,127], and the evidence base for psychoeducational interventions in LMICs remains limited [129].

Ninth, the risk-of-bias distribution (31.7% low risk, 41.9% moderate, 26.3% high risk) indicates that most included studies had methodological limitations. Low-risk-of-bias studies showed a marginally lower favorable rate (96.6%) in the direction-of-effect synthesis, consistent with more rigorous designs yielding more conservative estimates.

Tenth, the English-language restriction may have excluded relevant evidence from non-English-speaking regions, particularly in community-based and culturally adapted interventions where local-language publications may predominate.

### 4.10. Future Research Directions

The findings and limitations of this systematic review and meta-analysis point to several priority directions for future research, informed by recent advances in neuropsychology, digital technology, cultural adaptation, and implementation science [129,130,131,132,133,134,135,136,137,138,139,140,141].

First, individual participant data (IPD) meta-analyses [128] represent the most direct path to validating the moderator-based personalization framework proposed here. IPD approaches enable individual-level prediction models that overcome the ecological fallacy inherent in study-level moderator analyses and can identify interaction effects between participant characteristics and intervention features.

Second, prospective moderator studies should be designed to test the hypothesized predictors (baseline severity, duration, delivery format, theoretical framework) a priori rather than post hoc, with adequate statistical power for detecting moderator × treatment interactions. Pre-registration of moderator hypotheses is essential to distinguish confirmatory from exploratory findings.

Third, future primary studies should prioritize complete reporting of effect size data. Only 28.5% of included studies reported sufficient statistical data to compute standardized effect sizes, substantially limiting the quantitative meta-analytic base. Adoption of reporting guidelines (e.g., CONSORT, TIDieR) and routine reporting of means, standard deviations, and between-group comparisons would strengthen future meta-analytic synthesis.

Fourth, Sequential Multiple Assignment Randomized Trial (SMART) designs [124] offer a rigorous framework for adaptive intervention optimization, enabling empirical evaluation of sequential decision rules for personalized intervention delivery (e.g., “if insufficient response after 4 weeks of digital delivery, augment with human support”).

Fifth, external validation studies are needed to test whether moderator-based matching (e.g., assigning high-severity individuals to indicated programs or matching individuals with anxiety to mindfulness-based programs) improves individual outcomes compared with standard assignment [129,130,131]. The Personalized Advantage Index (PAI) methodology [75] provides a framework for such studies.

Sixth, machine learning and artificial intelligence approaches [73] should be applied to develop and validate prediction algorithms for differential treatment response. Recent advances in AI-biomarker integration and digital twin cognition frameworks [132] suggest that neurophysiological markers, including EEG-derived cognitive and affective biomarkers [133,137], may complement self-report moderators in predicting individual treatment response, potentially enabling real-time, biologically informed personalization that moves beyond the study-level moderators examined in the present review.

Seventh, mechanistic dismantling studies are needed to isolate which specific intervention components drive effects for which individuals, moving beyond the “package-level” comparisons that dominate the current evidence base. Neuropsychological research linking cognitive dynamics to anxiety disorders [134,138] and psychotic spectrum conditions [135,139] provides a foundation for identifying neurocognitive mechanisms underlying differential treatment response, which could inform component-level optimization.

Eighth, research in underrepresented populations—including individuals with severe mental illness, ethnic minorities, older adults, and LMIC settings—is needed to establish whether the moderator patterns identified here generalize across diverse contexts [126,127,128].

Ninth, innovative delivery modalities, including gamification of psychoeducational content within school settings [136,140] and co-produced digital tools developed iteratively with end-users for specific clinical populations [141], represent promising extensions of the conventional intervention formats examined in this review. Gamified approaches that integrate behavioral change techniques with interactive game mechanics may enhance engagement and skill acquisition among younger populations, while co-production methodologies ensure that digital interventions align with the lived experiences and preferences of target users.

Tenth, emerging interdisciplinary research bridging neuropsychology, digital technology, and health promotion offers promising foundations for next-generation psychoeducational interventions. Neuropsychological investigations of cognitive and affective dynamics in anxiety [134] and psychotic spectrum conditions [135] are increasingly informing the design of targeted intervention components that address specific neurocognitive deficits, an approach that aligns with the precision mental health paradigm advocated in the present review. Simultaneously, gamified health promotion programs that integrate neuropsychological principles with behavioral change techniques have shown preliminary effectiveness in educational settings [136], suggesting that interactive, technology-mediated delivery may enhance engagement across diverse populations. These converging lines of research—from neurocognitive profiling to gamified implementation—underscore the potential for psychoeducational interventions to evolve beyond conventional didactic formats toward adaptive, neurobiologically informed delivery systems [73,132].

**Table 5:** Proposed Evidence-Based Personalization Framework.

Phase	Decision Dimension	Personalization Recommendation	Supporting Evidence	Key References
Phase 1: Assessment & Selection	Baseline Severity	Assess symptom severity using validated instruments (e.g., PHQ-9, GAD-7). Classify per IOM framework: Universal (unselected, g = 0.45), Selective (at-risk, g = 0.53), or Indicated (elevated symptoms, g = 0.63). Allocate to stepped-care level accordingly.	Severity moderation: Q_M(2) = 9.86, *p* = 0.007 Indicated > Selective > Universal Stepped pattern confirmed	[100] van Straten [119] Cuijpers [75] DeRubeis
	Delivery Preferences	Assess individual preferences for format (face-to-face, digital, hybrid). Non-significant format difference (*p* = 0.118) supports honoring preferences. Preference-matched delivery associated with larger effects (g = 0.65 vs. 0.49).	Format moderation: Q_M = 4.28, *p* = 0.118 (ns) Preference matching: Q_M = 4.28, *p* = 0.039 *	[91] Elwyn [92] Lindhiem [93] Swift
	Modality Matching	Match modality to presenting concerns: Mindfulness for anxiety/stress reduction (g = 0.78/0.56); Positive psychology for well-being enhancement (g = 0.89) and positive affect (g = 0.72). Rumination predicts mindfulness benefit (β = 0.24); values-discrepancy predicts PP benefit (β = 0.19).	RQ4 comparison: Q_M(1) = 2.84, *p* = 0.092 (ns) Rumination: β = 0.24, *p* = 0.003 Values: β = 0.19, *p* = 0.002	[49] Khoury [50] Goldberg [52] Bolier [82] Gu
Phase 2: Program Design & Training	Theoretical Framework	Select theory-based programs (g = 0.57) over atheoretical approaches (g = 0.43). Prioritize interventions grounded in validated frameworks: HBM [26], SCT [27], TTM [28], CBT/ACT [83], mindfulness [82], or positive psychology [52].	Theory moderation: Q_M = 7.24, *p* = 0.007 * Δg = 0.14 (theory advantage)	[121] Webb [122] Michie [26,27,28]
	Implementation Fidelity	Invest in facilitator training and fidelity monitoring. Implementation fidelity positively associated with effect size (β = 0.14, *p* = 0.001). Teacher-delivered vs. external facilitator effects comparable (g = 0.52 vs. 0.56, *p* = 0.489), supporting task-sharing models.	Fidelity–outcome: β = 0.14, SE = 0.04, *p* = 0.001 Facilitator type: ns	[129] Singla [118] Kazdin
Phase 3: Personalized Implementation	Duration & Dosage	Plan programs for >8 weeks where feasible (g = 0.59 vs. 0.49 for ≤8 weeks). Median duration in effective programs = 10 weeks; range 4–16 weeks. Longer programs allow skill consolidation and behavioral practice.	Duration moderation: Q_M = 5.92, *p* = 0.015 * Δg = 0.10 (duration advantage)	[27] Bandura [84] Bandura [101] Anderson
	Digital Delivery	For digital delivery, use guided formats with human support (g = 0.59) rather than fully automated programs (g = 0.39). Implement supportive accountability: regular check-ins, progress feedback, motivational prompting. Consider JITAIs for real-time adaptation.	Guided vs. automated: Q_M = 5.14, *p* = 0.023 * Δg = 0.20	[102] Mohr [85] Lattie [87] Nahum-Shani [120] Linardon
	Clinical Integration	For clinical/vulnerable populations (RQ5), integrate into existing healthcare pathways (g = 0.60 vs. 0.46 for standalone). Group-based delivery advantageous when social support is low (β = −0.16, *p* = 0.001). Cultural adaptation enhances outcomes (Q_M = 4.12, *p* = 0.042).	Integration: Q_M = 3.98, *p* = 0.046 * Social support: β = −0.16 Cultural adapt.: *p* = 0.042 *	[123] Norcross [130] Benish [131] Bernal [129] Singla
Phase 4: Evaluation & Optimization	Outcome Monitoring	Use validated instruments for ongoing monitoring (e.g., ORS, PHQ-9). Low-ROB benchmark: g = 0.47 [0.41, 0.53]. Active-control benchmark: g = 0.43 [0.37, 0.49]. Expect non-specific factors contribute ~0.16 to waitlist-controlled estimates.	Low-ROB sensitivity: g = 0.47 [0.41, 0.53] Active-control: g = 0.43 [0.37, 0.49]	[110] Higgins [116] Guyatt [99] Wampold
	Booster & Maintenance	Schedule booster sessions post-intervention (g = 0.49 with boosters vs. 0.38 without, *p* = 0.027). Monitor for relapse and adapt intervention parameters based on individual response trajectory. Consider SMART designs for sequential optimization.	Booster effect: g = 0.49 vs. 0.38, *p* = 0.027 * Follow-up: 87 studies (46.8%)	[124] Collins [73] Chekroud [128] Riley
Caveats		This framework is a hypothesis-generating heuristic, NOT a validated clinical decision tool. Moderator models explained only R^2^ = 9–14% of total heterogeneity; 86–91% of variance remains attributable to unmeasured factors. Prospective validation required before prescriptive application. Ecological fallacy applies: study-level associations may not hold at the individual level.	R^2^ = 9–14% PI [0.05, 1.05] Ecological fallacy applies [74]	[74] Fisher [75] DeRubeis [128] Riley

Note. All direction-of-effect percentages from vote-counting across k = 186 studies following SWiM guidelines. Quantitative g values from DerSimonian–Laird random-effects meta-analysis of k = 53 studies with extractable effect sizes. Reference numbers correspond to the manuscript reference list. * Statistically significant at *p* < 0.05.

### 4.11. Implementation Framework

The direction-of-effect patterns reported in paragraph 3.8 (Table 5; Figure 9), the quantitative meta-analytic results (Table 3), and the contextual patterns identified across the five research questions collectively point toward an integrated framework for translating this evidence into practice. While the preceding Discussion subsections have addressed the individual findings, their clinical utility ultimately depends on how they can be assembled into a coherent, sequential decision process. To this end, we propose a preliminary, multi-phase implementation framework grounded in converging quantitative and direction-of-effect evidence and organized into four phases. The framework is presented in Table 5 and as a visual flow diagram in Figure 12.

*Phase 1: Assessment and Selection.* Informed by the baseline severity gradient (Universal 95.6% → Selective 98.6% → Indicated 100% favorable, with the largest quantitative effect in clinical populations: g = 0.91 [0.26, 1.56]), this phase involves standardized baseline assessment using validated instruments to classify individuals along the IOM prevention continuum (universal, selective, indicated) and guide stepped-care allocation [100]. Assessment should also capture delivery format preferences [91,92,93] and presenting concerns to inform modality matching (mindfulness for anxiety/stress; positive psychology for well-being).

*Phase 2: Program Design and Facilitator Training.* Informed by the higher favorable rate for theory-based interventions (98.2% vs. 95.2%) and the consistent finding across settings that theoretically grounded programs produce positive outcomes, this phase emphasizes selection of evidence-based, theoretically grounded programs [121,122] and investment in facilitator competency and implementation fidelity monitoring.

*Phase 3: Personalized Implementation.* Informed by the duration pattern (>8 weeks: 99.0% favorable vs. ≤8 weeks: 96.6%), the descriptive advantage of guided digital delivery, and the consistent effectiveness of interventions integrated into existing care pathways. Programs should be planned for ≥8 weeks where feasible, digital delivery should include human support, and clinical/vulnerable population programs should integrate into existing healthcare structures.

*Phase 4: Evaluation and Optimization.* Ongoing outcome monitoring using validated measures enables adaptive modification of intervention parameters. The leave-one-out analysis (g = 0.61–0.67) confirms that the quantitative finding is stable and not driven by any single study. This phase aligns with the adaptive intervention paradigm [124] and the SMART design framework for sequential optimization.

The term “personalized” in this framework denotes evidence-informed stratification—selecting among intervention options based on empirically identified moderator patterns—rather than algorithmic individual-level prediction. This distinction is critical: the present findings identify population-level associations (e.g., the severity gradient, duration effects, delivery format equivalence) that inform rational intervention selection, but the ecological fallacy applies to all such study-level associations [74]. The complete four-phase framework, presented visually in Figure 12, should therefore be viewed as a preliminary, hypothesis-generating heuristic rather than a validated prescriptive tool. The quantitative meta-analysis is based on k = 53 studies with extractable effect sizes—a subset of the full evidence base—and while the direction-of-effect synthesis across all 186 studies provides convergent support, study-level associations cannot be directly extrapolated to individual-level treatment decisions. The critical next step is to evaluate whether moderator-informed matching, as operationalized in this framework, produces superior individual outcomes compared to standard non-personalized assignment, using prospective designs such as individual participant data meta-analyses [128], SMART trials [124], or Personalized Advantage Index studies [75].

## 5. Conclusions

This systematic review and meta-analysis of 186 studies across more than 30 countries found that psychoeducational interventions were associated with significant, meaningful improvements in psychological well-being across educational, community, and clinical settings. The quantitative meta-analysis of 53 studies with extractable effect sizes yielded a medium-to-large pooled effect, and the direction-of-effect synthesis confirmed near-universal effectiveness, with 182 of 186 studies (97.8%) reporting favorable outcomes. This convergence between methodologically distinct analytical approaches strengthens confidence in the overall positive conclusion.

Consistent positive effects were observed across all intervention types—including acceptance and commitment therapy, positive psychology, mindfulness-based interventions, social-emotional learning, and health education. The largest quantitative effects were observed in clinical and vulnerable populations, followed by university programs, school-based interventions, mindfulness/positive psychology, and community-based programs.

The direction-of-effect synthesis identified several patterns that could inform the selection of personalized interventions. Baseline symptom severity showed a stepwise gradient in favorable outcomes (indicated: 100%, selective: 98.6%, universal: 95.6%), reinforced by the quantitative finding that clinical populations yielded the largest pooled effect. Programs exceeding eight weeks showed marginally higher favorable rates than shorter programs, and theory-based interventions outperformed atheoretical programs in the proportion of favorable outcomes. Digital interventions produced comparable favorable rates to face-to-face delivery, supporting patient preference-guided format selection and scalable implementation through guided digital platforms.

These findings carry implications for clinical practice, public health policy, and educational programming. Psychoeducational interventions may be integrated into universal prevention programs, with stepped-care models allocating more intensive resources to individuals with higher severity. For clinical populations, including patients with chronic illnesses, caregivers, refugees, and perinatal women, integration into existing care pathways may be considered. Comparable effectiveness across delivery formats supports policy investments in digital infrastructure, particularly in underserved communities.

Based on these findings, the study proposes a preliminary four-phase implementation framework encompassing: individualized assessment informed by the baseline severity gradient; facilitator training emphasizing theoretical grounding; personalized implementation with duration optimization and guided digital delivery; and outcome evaluation with adaptive modification. This framework should be understood as a hypothesis-generating guide requiring prospective validation before prescriptive application.

Several limitations must be acknowledged. Only 53 of 186 included studies reported sufficient data for quantitative meta-analysis; the remaining 133 studies were synthesized through direction-of-effect classification. The high heterogeneity in the quantitative subset (I^2^ = 96.1%) and wide prediction interval [−0.46, 1.78] indicate limited generalizability of the pooled estimate as a single summary measure. The ecological fallacy applies to all study-level associations. Additional limitations include a predominant reliance on self-reported outcomes, a risk-of-bias distribution indicating that most studies had methodological limitations, an English-language restriction, and limited long-term follow-up data.

Future research should focus on individual participant data meta-analyses enabling individual-level prediction; prospective moderator studies with a priori hypotheses; complete effect size reporting in primary studies; adaptive trial designs such as SMART; machine learning approaches for treatment matching; dismantling studies isolating active intervention components; expansion to underrepresented populations; and implementation studies evaluating the real-world feasibility of personalized selection frameworks.

Psychoeducational interventions offer an evidence-based approach to addressing the growing global burden of mental health problems. The convergence of a medium-to-large quantitative effect with a near-universal favorable direction across 186 diverse studies provides a preliminary empirical rationale for personalized intervention selection, suggesting the potential to move beyond one-size-fits-all models toward precision mental health approaches that match intervention characteristics to individual needs, preferences, and clinical profiles. The critical next step is the transition from study-level associations, which remain subject to the ecological fallacy, to validated individual-level prediction algorithms through prospective research.

## Figures and Tables

**Table 1:** Eligibility Criteria for Study Inclusion and Exclusion (PICOS Framework).

PICOS Element	Inclusion Criteria	Exclusion Criteria
Population	Children, adolescents, or adults of any age; any clinical or non-clinical setting; no restrictions on baseline symptom severity; both community and clinical populations	Animal studies; studies with no human participants
Intervention	Structured psychoeducational programs explicitly designed to promote psychological well-being, mental health literacy, or psychosocial functioning; programs with identifiable content (curriculum, modules, sessions); any delivery format (face-to-face, digital, hybrid); any theoretical framework (CBT, ACT, mindfulness, positive psychology, SEL, etc.); duration ≥ 4 weeks with ≥2 structured sessions	Purely pharmacological interventions; single-session workshops or one-off seminars; interventions with no psychoeducational component; programs < 4 weeks or <2 sessions
Comparison	Any comparator: waitlist, no-treatment, treatment-as-usual, attention control, or active control; studies with at least one comparison group	Single-group studies without any comparison condition; case studies or case series
Outcomes	At least one validated measure of psychological well-being, mental health, or related psychosocial outcomes; validated instruments (e.g., PHQ-9, GAD-7, WEMWBS, DASS-21, WHO-5, PSS, SWLS); quantitative outcome data permitting effect size calculation or direction-of-effect classification	Studies measuring only academic, physical health, or purely behavioral outcomes without a psychological component; studies reporting only qualitative findings
Study Design	Randomized controlled trials (RCTs); cluster-randomized trials; quasi-experimental designs with a comparison group; controlled pre-post designs	Uncontrolled pre-post studies; cross-sectional surveys; systematic reviews or meta-analyses; protocols, commentaries, editorials; secondary analyses of previously included datasets
Additional Criteria	Published January 2015–December 2024 (including early online publications); published in English; peer-reviewed journal articles; sufficient data for Hedges’ g calculation (for quantitative meta-analytic inclusion) or quantitative outcomes reported permitting direction-of-effect classification (for narrative synthesis inclusion)	Published before January 2015; non-English publications; grey literature, dissertations, conference abstracts; duplicate or overlapping datasets (most comprehensive report retained)

Note. PICOS = Population, Intervention, Comparison, Outcomes, Study Design. A two-tier inclusion approach was applied: studies providing sufficient data for standardized effect size calculation (Hedges’ g) were included in the quantitative meta-analysis (k = 53); studies meeting all inclusion criteria but reporting outcomes in formats not convertible to Hedges’ g were included in the direction-of-effect narrative synthesis (k = 133); together these constitute the 186 included studies (N = 50,328 verified from 124 studies reporting sample sizes). An additional 24 studies that met all inclusion criteria but lacked any usable quantitative outcome data were excluded from the review (total screened at the full-text stage: *n* = 210; see Figure 1). Characteristics of all included studies are presented in Supplementary Table S1; the complete study-level dataset is provided in Supplementary Table S2.

**Table 3:** Summary of Two-Tier Evidence Synthesis by Research Question.

**Panel A: Quantitative Meta-Analysis (Studies with Extractable Effect Sizes)**													
**Research Question**	**k (Quant)**	**k** **(Total)**	**N** **(Verified)**		**g**	**95% CI**		**I^2^ (%)**	**τ^2^**		**Q (df)**		**95% PI**
RQ1: School-Based	15	61	26,548 (38)		0.60	[0.24, 0.96]		97.2	0.481		501.18 (14)		[−0.63, 1.82]
RQ2: University-Based	16	42	5285 (32)		0.62	[0.39, 0.85]		89.8	0.182		147.11 (15)		[−0.24, 1.49]
RQ3: Community-Based	5	23	3087 (14)		0.49	[0.28, 0.71]		36.3	0.021		6.28 (4)		[0.13, 0.85]
RQ4: Mindfulness/PP	10	22	8366 (13)		0.55	[0.33, 0.76]		87.3	0.078		71.11 (9)		[−0.04, 1.00]
RQ5: Clinical/Vulnerable	7	38	7042 (27)		0.91	[0.26, 1.56]		98.1	0.748		310.52 (6)		[−0.90, 2.73]
Overall	53	186	50,328 (124)		0.66	[0.50, 0.82]		96.1	0.322		1324.15 (52)		[−0.46, 1.78]
**Panel B: Direction-of-Effect Narrative Synthesis (all included Studies)**													
**Research Question**	**k**	**Positive**		**Mixed**			**Null**			**Unclear**		**Favorable**	
RQ1: School-Based	61	42 (69%)		18 (30%)			0 (0%)			1 (2%)		60/61 (98%)	
RQ2: University-Based	42	29 (69%)		13 (31%)			0 (0%)			0 (0%)		42/42 (100%)	
RQ3: Community-Based	23	17 (74%)		4 (17%)			0 (0%)			2 (9%)		21/23 (91%)	
RQ4: Mindfulness/PP	22	16 (73%)		6 (27%)			0 (0%)			0 (0%)		22/22 (100%)	
RQ5: Clinical/Vulnerable	38	27 (71%)		10 (26%)			0 (0%)			1 (3%)		37/38 (97%)	
Overall	186	131 (70%)		51 (27%)			0 (0%)			4 (2%)		182/186 (98%)	

Note. Panel A: g = Hedges’ g (random-effects pooled estimate using DerSimonian–Laird method) [109]; CI = confidence interval; PI = 95% prediction interval [115]; I^2^ = proportion of variance due to heterogeneity [110]; τ^2^ = between-study variance; Q = Cochran’s Q statistic. All pooled effects are statistically significant at *p* < 0.001. N (verified) = sum of sample sizes extractable from the primary database, with number of reporting studies in parentheses. Effect sizes computed from Cohen’s d, F statistics, t statistics, odds ratios, or regression coefficients reported in the original studies. Panel B: Direction classified from reported findings, intervention effects, and study summaries. Positive = statistically significant benefit on ≥1 primary outcome; Mixed = some outcomes significant, others not; Null = no significant effects; Unclear = direction not determinable.

**Table 4:** Direction-of-Effect Moderator Analysis: Key Personalization Variables (k = 186).

Moderator Variable	Category/Level	k	Favorable	% Favorable
Intervention Duration ^a^	≤8 weeks	89	86/89	96.6%
	>8 weeks	97	96/97	99.0%
Theoretical Framework	Theory-based	165	162/165	98.2%
	Atheoretical	21	20/21	95.2%
Baseline Severity (IOM)	Universal	68	65/68	95.6%
	Selective	72	71/72	98.6%
	Indicated	46	46/46	100%
Control Condition	Active control	64	62/64	96.9%
	Waitlist/No treatment	122	120/122	98.4%

Note. Direction-of-effect vote-counting across all 186 included studies following SWiM guidelines. Favorable = studies reporting positive or mixed results (positive: statistically significant benefit on ≥1 primary outcome; mixed: some outcomes significant, others not). Direction classified from study summaries, findings, and intervention effects columns in the primary database. Moderator k-values verified against Supplementary Tables: duration (89 + 97 = 186), framework (165 + 21 = 186), severity (68 + 72 + 46 = 186), and control condition (64 + 122 = 186). Study design, risk of bias, delivery format, and per-RQ direction-of-effect distributions are reported in Table 2 and are not duplicated here. ^a^ Duration categories from original systematic coding; ≤8 weeks and >8 weeks defined by the reported intervention period. The stepped severity gradient (95.6% → 98.6% → 100%) and the theory-based advantage (98.2% vs. 95.2%) inform Phase 1 and Phase 2 of the proposed implementation framework.

## Data Availability

The study-level dataset supporting this meta-analysis is available in Supplementary Tables S1 and S2. The review protocol is registered on the Open Science Framework (OSF) at osf.io/aunks (DOI: 10.17605/OSF.IO/AUNKS). Individual participant data from primary studies are not available, as this review exclusively analyzed published aggregate-level data.

## Supplementary Materials

The following supporting information can be downloaded at: https://www.mdpi.com/article/10.3390/jpm16040215/s1, Table S1: Individual Study Characteristics of 186 Included Studies with Tier Classification, Direction of Effect, Study Design, and Quantitative Effect Sizes (k = 53 Subset); Table S2: Extended Study-Level Data with Study Objectives, Main Findings, Intervention Effects, Outcome Instruments, Key Statistics, and Hypotheses Tested; Table S3: Moderator Variable Definitions, Coding Rules, and Inter-Rater Agreement; Table S4: Study-Level Risk-of-Bias Assessment Matrix (k = 186); Table S5: GRADE Summary of Findings for Each Research Question; Table S6: Sensitivity and Influence Diagnostic Results (k = 53); Table S7: PRISMA 2020 Checklist.

## Abbreviations

The following abbreviations are used in this manuscript:

AAQ-II	Acceptance and Action Questionnaire-II
ACT	Acceptance and Commitment Therapy/Training
AI	Artificial Intelligence
C-RCT	Cluster-Randomized Controlled Trial
CAMM	Child and Adolescent Mindfulness Measure
CBT	Cognitive Behavioral Therapy
CD-RISC	Connor-Davidson Resilience Scale
CI	Confidence Interval
CMA	Comprehensive Meta-Analysis
DASS-21	Depression Anxiety Stress Scales-21
DOI	Digital Object Identifier
EMA	Ecological Momentary Assessment
ERIC	Education Resources Information Center
FFMQ	Five-Facet Mindfulness Questionnaire
FS	Flourishing Scale
g	Hedges’ g (effect size)
GAD-7	Generalized Anxiety Disorder 7-item Scale
GHQ-12	General Health Questionnaire-12
GRADE	Grading of Recommendations Assessment, Development and Evaluation
GSE	General Self-Efficacy Scale
HBM	Health Belief Model
I^2^	I-squared (heterogeneity statistic)
IPD	Individual Patient Data
ITT	Intention-to-Treat
JBI	Joanna Briggs Institute
JITAI	Just-in-Time Adaptive Intervention
k	Number of studies
K10	Kessler Psychological Distress Scale
M	Mean
MAAS	Mindful Attention Awareness Scale
MBI	Mindfulness-Based Intervention
MBSR	Mindfulness-Based Stress Reduction
Mdn	Median
MeSH	Medical Subject Headings
MHC-SF	Mental Health Continuum-Short Form
mHealth	Mobile Health
*n*	Number (subset/subgroup)
N	Total number of participants
NNT	Number Needed to Treat
NOS	Newcastle–Ottawa Scale
OSF	Open Science Framework
*p*	Probability value (*p*-value)
PANAS	Positive and Negative Affect Schedule
PERMA	PERMA Profiler (Positive Emotion, Engagement, Relationships, Meaning, Accomplishment)
PHQ-9	Patient Health Questionnaire-9
PI	Prediction Interval
PP	Positive Psychology
PRISMA	Preferred Reporting Items for Systematic Reviews and Meta-Analyses
PSS	Perceived Stress Scale
PWB	Psychological Well-Being Scale
Q	Cochran’s Q (heterogeneity test statistic)
QM	Omnibus test of moderator
R^2^	R-squared (coefficient of determination)
RCT	Randomized Controlled Trial
REDCap	Research Electronic Data Capture
REML	Restricted Maximum Likelihood
RoB 2.0	Risk of Bias Tool 2.0 (Cochrane)
RQ	Research Question
SE	Standard Error
SEL	Social–Emotional Learning
SF-36	36-Item Short Form Health Survey
SMART	Sequential Multiple Assignment Randomized Trial
SOC-13	Sense of Coherence Scale-13
SWLS	Satisfaction with Life Scale
WEMWBS	Warwick–Edinburgh Mental Well-Being Scale
WHO-5	World Health Organization-Five Well-Being Index
β	Beta coefficient (regression)
κ	Cohen’s Kappa (inter-rater reliability)
τ^2^	Tau-squared (between-study variance)
