# Supplementary material for: Toward Personalized Psychoeducational Interventions for Psychophysical Health: A Systematic Review and Meta-Analysis for Tailored Intervention Selection
Source: J Pers Med. 2026 Apr 14;16(4):215. doi: 10.3390/jpm16040215 (PMC13117039; doi:10.3390/jpm16040215)
Supplement: Supplementary file 1 [file jpm-16-00215-s001.zip › Table_S7_PRISMA_2020_Checklist.pdf]

PRISMA 2020 Checklist

Section and Topic	Item #	Checklist item	Location where item is reported
**TITLE Toward Personalized Psychoeducational Interventions for Psychophysical Health: A Systematic Review and Meta-Analysis for Tailored Intervention Selection**			
Title	1	Identify the report as a systematic review.	Title page; line 1 ("Systematic Review") and full title
**ABSTRACT**			
Abstract	2	See the PRISMA 2020 for Abstracts checklist.	Abstract section (structured abstract with Background, Methods, Results, Conclusions)
**INTRODUCTION**			
Rationale	3	Describe the rationale for the review in the context of existing knowledge.	§1.1–1.7. Establishes gaps in personalized intervention selection, reviews prior meta-analyses [26–38], describes limitations of single-modality reviews, and provides conceptual justification for grouping diverse modalities under a psychoeducational framework (§1.4: common core mechanism, psychological literacy, cross-modality comparison).
Objectives	4	Provide an explicit statement of the objective(s) or question(s) the review addresses.	§1.8. Five research questions (RQ1–RQ5) explicitly stated, with corresponding hypotheses. RQ1: school-based; RQ2: university-based; RQ3: community-based; RQ4: mindfulness/positive psychology; RQ5: clinical/vulnerable populations.
**METHODS**			
Eligibility criteria	5	Specify the inclusion and exclusion criteria for the review and how studies were grouped for the syntheses.	§2.2. PICOS framework in Table 1. Inclusion: peer-reviewed, 2015–2024, English, psychoeducational interventions, quantitative outcomes. Exclusion: reviews, protocols, qualitative-only, pharmacological-only, non-peer-reviewed. Two-tier grouping: quantitative meta-analysis (k = 53) and direction-of-effect narrative synthesis (k = 133).
Information sources	6	Specify all databases, registers, websites, organisations, reference lists and other sources searched or consulted to identify studies. Specify the date when each source was last searched or consulted.	§2.3. Seven databases: PubMed/MEDLINE, PsycINFO, Scopus, Web of Science, ERIC, Cochrane Library, Google Scholar. Reference lists of included studies. OSF preregistration (osf.io/aunks).
Search strategy	7	Present the full search strategies for all databases, registers and websites, including any filters and limits used.	§2.3 and Appendix A (Supplementary Materials). Boolean search strings with MeSH terms and free-text terms. Date filter: January 2015–December 2024. Language: English.
Selection process	8	Specify the methods used to decide whether a study met the inclusion criteria of the review, including how many reviewers screened each record and each report retrieved, whether they worked independently, and if applicable, details of automation tools used in the process.	§2.4. Two independent reviewers screened titles/abstracts and full texts using standardized forms. Disagreements resolved by consensus or third reviewer. Inter-rater reliability: Cohen's $\kappa \geq 0.85$ across all coding categories (Supplementary Table S3).
Data collection process	9	Specify the methods used to collect data from reports, including how many reviewers collected data from each report, whether they worked independently, any processes for obtaining or confirming data from study investigators, and if applicable, details of automation tools used in the process.	§2.4. Standardized data extraction form. Two independent coders. Corresponding authors contacted for missing data. Extraction included study design, sample size, intervention characteristics, outcome measures, effect sizes, moderator variables.
Data items	10a	List and define all outcomes for which data were sought. Specify whether all results that were compatible with each outcome domain in each study were sought (e.g. for all measures, time points, analyses), and if not, the methods used to decide which results	§2.2.1. Primary outcomes: psychological well-being, mental health, psychosocial functioning. Outcome instruments listed in Supplementary Table S2. For studies reporting multiple outcomes,

Section and Topic	Item #	Checklist item	Location where item is reported
		to collect.	the primary well-being measure was selected; if not designated, the most commonly used validated instrument was prioritized.
	10b	List and define all other variables for which data were sought (e.g. participant and intervention characteristics, funding sources). Describe any assumptions made about any missing or unclear information.	§2.4 and Supplementary Table S3. Variables: study design, population, country, sample size, intervention type, theoretical framework, duration, delivery format (face-to-face/digital/hybrid), IOM prevention level (universal/selective/indicated), control condition, risk of bias ratings.
Study risk of bias assessment	11	Specify the methods used to assess risk of bias in the included studies, including details of the tool(s) used, how many reviewers assessed each study and whether they worked independently, and if applicable, details of automation tools used in the process.	§2.5. RoB 2.0 for RCTs; Newcastle-Ottawa Scale and JBI checklist for non-randomized designs. Two independent reviewers. Domain-level and summary-level ratings documented in Supplementary Tables S3 and S4 ($k = 186$ matrix). Inter-rater $\kappa = 0.85$.
Effect measures	12	Specify for each outcome the effect measure(s) (e.g. risk ratio, mean difference) used in the synthesis or presentation of results.	§2.6. Hedges' g with 95% CI for the quantitative subset ($k = 53$). Direction-of-effect classification (positive/mixed/null/unclear) for all 186 studies following SWiM guidelines [104].
Synthesis methods	13a	Describe the processes used to decide which studies were eligible for each synthesis (e.g. tabulating the study intervention characteristics and comparing against the planned groups for each synthesis (item #5)).	§2.5–2.6. Studies with extractable effect size data (means, SDs, sample sizes, or test statistics convertible to Hedges' g) included in quantitative meta-analysis ($k = 53$, 28.5%). Remaining 133 studies included in direction-of-effect narrative synthesis. 24 studies excluded for meeting exclusion criteria at eligibility stage.
	13b	Describe any methods required to prepare the data for presentation or synthesis, such as handling of missing summary statistics, or data conversions.	§2.6. Effect sizes calculated as Hedges' g (small-sample bias correction). When not directly reported, computed from means/SDs, t-statistics, F-statistics, or p-values with sample sizes. SE estimated as $\sqrt{(4/N + g^2/2N)}$ where needed.
	13c	Describe any methods used to tabulate or visually display results of individual studies and syntheses.	§2.6; Figures 1–12. Forest plot (Figure 2, $k = 53$), direction-of-effect bar charts (Figures 3–7), cross-RQ heatmap (Figure 8), moderator summary (Figure 9), ROB summary (Figure 10), funnel plot (Figure 11), implementation framework (Figure 12). Tables 1–5 and Supplementary Tables S1–S6.
	13d	Describe any methods used to synthesize results and provide a rationale for the choice(s). If meta-analysis was performed, describe the model(s), method(s) to identify the presence and extent of statistical heterogeneity, and software package(s) used.	§2.6. DerSimonian–Laird random-effects model. Q statistic, I^2, τ^2, and 95% prediction intervals for heterogeneity. Direction-of-effect vote-counting following SWiM guidelines [104]. Software: R/metafor package.
	13e	Describe any methods used to explore possible causes of heterogeneity among study results (e.g. subgroup analysis, meta-regression).	§2.6. Subgroup analyses by RQ (five settings). Moderator analyses: study design, theoretical framework, IOM prevention level, intervention duration, delivery format, control condition. Direction-of-effect cross-tabulation across all 186 studies.
	13f	Describe any sensitivity analyses conducted to assess robustness of the synthesized results.	§2.6 and §3.10. Leave-one-out analysis ($k = 53$, range: $g = 0.61$–0.67). Cook's distance and studentized residuals. Concordance between quantitative and direction-of-effect findings. Results in Supplementary Table S6.
Reporting bias assessment	14	Describe any methods used to assess risk of bias due to missing results in a synthesis (arising from reporting biases).	§2.6 and §3.9. Funnel plot visual inspection (Figure 11). Egger's regression test (intercept = −0.18, non-significant). Rosenthal's fail-safe N = 22,942 (criterion = 275). Trim-and-fill analysis.

PRISMA 2020 Checklist

Section and Topic	Item #	Checklist item	Location where item is reported
Certainty assessment	15	Describe any methods used to assess certainty (or confidence) in the body of evidence for an outcome.	§2.6 and Supplementary Table S5. GRADE framework applied to each of the five research questions. Domains assessed: risk of bias, inconsistency, indirectness, imprecision, publication bias. Ratings: Moderate for RQ1, RQ2, RQ4, RQ5; Low for RQ3.
**RESULTS**			
Study selection	16a	Describe the results of the search and selection process, from the number of records identified in the search to the number of studies included in the review, ideally using a flow diagram.	§3.1 and Figure 1 (PRISMA 2020 flow diagram). 2,847 records identified → 955 duplicates removed → 1,892 screened → 465 full-text assessed → 255 excluded → 210 met criteria → 186 included (53 quantitative + 133 narrative) + 24 excluded at eligibility.
	16b	Cite studies that might appear to meet the inclusion criteria, but which were excluded, and explain why they were excluded.	§3.1 and §2.2.2. 255 excluded for: not meeting intervention criteria (89), wrong outcomes (67), insufficient statistics (42), secondary syntheses (24), inadequate design (18), duplicates (15). 24 further excluded for protocols/qualitative/no usable data.
Study characteristics	17	Cite each included study and present its characteristics.	Table 2 (summary characteristics by RQ); Supplementary Table S1 (individual study characteristics for all 186 studies: ref, authors, RQ, tier, direction, design, intervention, outcomes, framework, population, N, g, CI, SE); Supplementary Table S2 (extended data with objectives, findings, instruments).
Risk of bias in studies	18	Present assessments of risk of bias for each included study.	§3.9 and Supplementary Table S4. Study-level ROB matrix for all 186 studies with domain-level ratings. Summary: Low 59 (31.7%), Moderate 78 (41.9%), High 49 (26.3%). Figure 10 (ROB summary).
Results of individual studies	19	For all outcomes, present, for each study: (a) summary statistics for each group (where appropriate) and (b) an effect estimate and its precision (e.g. confidence/credible interval), ideally using structured tables or plots.	Figure 2 (forest plot with individual study g, 95% CI, and weight for all k = 53 quantitative studies, labeled with Author (Year) [Ref]). Supplementary Table S1 (g, CI lower, CI upper, SE for each quantitative study). Direction-of-effect classification for all 186 studies.
Results of syntheses	20a	For each synthesis, briefly summarise the characteristics and risk of bias among contributing studies.	Table 2 (characteristics by RQ); §3.2–3.8 (per-RQ descriptions of contributing studies, designs, populations, settings). §3.9 (ROB distribution across quantitative subset).
	20b	Present results of all statistical syntheses conducted. If meta-analysis was done, present for each the summary estimate and its precision (e.g. confidence/credible interval) and measures of statistical heterogeneity. If comparing groups, describe the direction of the effect.	§3.2–3.8 and overall results section. Overall: $g = 0.66$ [0.50, 0.82], $Q(52) = 1{,}324.15$, $I^2 = 96.1\%$, $\tau^2 = 0.322$, PI [−0.46, 1.78]. Per-RQ: RQ1 $g = 0.60$, RQ2 $g = 0.62$, RQ3 $g = 0.49$, RQ4 $g = 0.55$, RQ5 $g = 0.91$. Direction: 182/186 (97.8%) favorable.
	20c	Present results of all investigations of possible causes of heterogeneity among study results.	§3.2–3.8 (per-RQ moderator patterns); §4.3 (moderator synthesis). Severity gradient: indicated 100%, selective 98.6%, universal 95.6%. Duration: >8 weeks 99.0% vs ≤8 weeks 96.6%. Theory-based 98.2% vs atheoretical 95.2%. Digital 97.8% vs face-to-face 97.1%.
	20d	Present results of all sensitivity analyses conducted to assess the robustness of the synthesized results.	§3.10. Leave-one-out: $g = 0.61$–0.67 (±0.04 max change). Cook's distance: no influential outliers. Direction concordance: quantitative ($g = 0.66$) and direction-of-effect (97.8% favorable) converge. Supplementary Table S6.
Reporting biases	21	Present assessments of risk of bias due to missing results (arising from reporting biases) for each synthesis assessed.	§3.9 and Figure 11. Funnel plot (k = 53): Egger's intercept = −0.18 (non-significant). Fail-safe N = 22,942 (83× the 5k+10 = 275

Section and Topic	Item #	Checklist item	Location where item is reported
			criterion). 28.5% reporting rate acknowledged as limitation.
Certainty of evidence	22	Present assessments of certainty (or confidence) in the body of evidence for each outcome assessed.	Supplementary Table S5 (GRADE). RQ1: Moderate (⊕⊕⊕○); RQ2: Moderate (⊕⊕⊕○); RQ3: Low (⊕⊕○○); RQ4: Moderate (⊕⊕⊕○); RQ5: Moderate (⊕⊕⊕○). Four of five RQs rated Moderate; RQ3 downgraded for indirectness.
**DISCUSSION**			
Discussion	23a	Provide a general interpretation of the results in the context of other evidence.	§4.1–4.6. Interprets findings relative to prior meta-analyses [26–38]. Discusses severity gradient, duration effects, delivery format equivalence, clinical implications. Contextualizes heterogeneity (§4.8): "genuine variation in true effect sizes" that the review "seeks to characterize rather than eliminate."
	23b	Discuss any limitations of the evidence included in the review.	§4.9. Only 28.5% provided extractable effect sizes. High heterogeneity (I^2 = 96.1%). Self-reported outcomes predominant. English-language restriction. Limited long-term follow-up. Risk-of-bias distribution. Ecological fallacy applies to all study-level moderator associations.
	23c	Discuss any limitations of the review processes used.	§4.9. Study-level moderator analyses cannot identify individual-level predictors. Direction-of-effect classification establishes consistency but not magnitude. Two-tier approach may over- or under-represent certain settings. Coding subjectivity despite high inter-rater reliability.
	23d	Discuss implications of the results for practice, policy, and future research.	§4.3–4.6 (clinical implications, stepped-care models, digital scalability); §4.10 (11 future research directions including IPD meta-analyses, SMART designs, PAI validation, machine learning, dismantling studies); §4.11 (four-phase implementation framework, Table 5, Figure 12).
**OTHER INFORMATION**			
Registration and protocol	24a	Provide registration information for the review, including register name and registration number, or state that the review was not registered.	§2.1. Open Science Framework (OSF): osf.io/aunks. DOI: 10.17605/OSF.IO/AUNKS.
	24b	Indicate where the review protocol can be accessed, or state that a protocol was not prepared.	§2.1. Protocol pre-registered with OSF (osf.io/aunks) prior to data extraction.
	24c	Describe and explain any amendments to information provided at registration or in the protocol.	§2.1 and §4.9. Original protocol specified six research questions (RQ1–RQ6); RQ5 (structured psychoeducation, k = 3) was consolidated into RQ3 due to insufficient studies, yielding five final RQs. Original target was k = 55 quantitative; corrected to k = 53 after identification of two duplicate papers.
Support	25	Describe sources of financial or non-financial support for the review, and the role of the funders or sponsors in the review.	Funding statement: "This research received no external funding."
Competing interests	26	Declare any competing interests of review authors.	Conflicts of Interest statement: "The authors declare no conflicts of interest."
Availability of data, code and other materials	27	Report which of the following are publicly available and where they can be found: template data collection forms; data extracted from included studies; data used for all analyses; analytic code; any other materials used in the review.	Data Availability Statement and Supplementary Materials. Tables S1–S6 contain all study-level data. OSF registration: osf.io/aunks. Individual study data in Supplementary Tables S1 (characteristics,

Section and Topic	Item #	Checklist item	Location where item is reported
			effect sizes) and S2 (extended data). Moderator coding in S3. ROB matrix in S4. GRADE in S5. Sensitivity in S6.

From: Page MJ, McKenzie JE, Bossuyt PM, Boutron I, Hoffmann TC, Mulrow CD, et al. The PRISMA 2020 statement: an updated guideline for reporting systematic reviews. BMJ 2021;372:n71. doi: 10.1136/bmj.n71.
This work is licensed under CC BY 4.0. To view a copy of this license, visit https://creativecommons.org/licenses/by/4.0/
